# Interactions Between Nutrition Professionals and Industry: A Scoping Review

**DOI:** 10.34172/ijhpm.2023.7626

**Published:** 2023-08-22

**Authors:** Virginie Hamel, Marita Hennessy, Mélissa Mialon, Jean-Claude Moubarac

**Affiliations:** ^1^Department of Nutrition, Faculty of Medicine, University of Montreal, Montreal, QC, Canada; ^2^Centre de recherche en Santé publique, Montreal, QC, Canada; ^3^College of Medicine and Health, University College Cork, Cork, Ireland; ^4^Trinity Business School, Trinity College Dublin, Dublin, Ireland

**Keywords:** Food Industry, Conflict of Interest, Dietitian, Nutritionist, Nutrition Professionals

## Abstract

**Background:** In recent years, interactions between nutrition professionals (NPs) and the food industry, such as sponsorship arrangements, have raised concerns, particularly as these may negatively impact the trustworthiness and credibility of the nutrition profession. This study aimed to map the literature and identify knowledge gaps regarding interactions between NPs and industry. We sought to examine the nature of such interactions and NPs perspectives about these, as well as the risks and solutions.

**Methods:** We conducted a scoping review according to a pre-registered protocol, searching eight electronic databases and grey literature sources in March 2021 to identify documents for inclusion. Two independent reviewers screened citations for inclusion and conducted data extraction. Quantitative and qualitative syntheses were conducted.

**Results:** We identified 115 documents for analysis, published between 1980 to 2021, with a majority from the United States (n=59, 51%). Only 32% (n=37) were empirical studies. The food industry was the most frequent industry type discussed (n=91, 79%). We identified 32 types of interactions between NPs and industry, such as continuing education provided by industry and sponsorship of professional bodies and health and nutrition organizations. The financial survival of nutrition organizations and continuing education access for NPs were the most frequently cited advantages of industry-NPs interactions. On the other hand, undermining public trust, NPs credibility and public health nutrition recommendations were pointed out as risks of these interactions. Following a code of ethics, policies, or guidelines was the most frequently proposed solution for managing these interactions.

**Conclusion:** Despite the increasing attention given to this issue, few empirical papers have been published to date. There is a need for more research to better and systematically document industry interactions with NPs and the impacts associated with these, as well as more research on effective management strategies.

**Registry Name and Number:** Interactions between nutrition professionals and industry actors: A scoping review protocol. doi:10.17605/OSF.IO/Q6PUA

## Background

 Diets high in ultra-processed foods (UPFs) are linked to poor health due to their low nutritional value.^1-3^ UPFs are industrial formulations made of refined substances, such as sugars, oil and starches, as well as additives, and contain little or no whole foods.^4^ Increasingly, these products are cited in dietary guidelines to reduce their consumption in the population.^5,6^ Public health researchers and advocates are also increasingly critical of the role of powerful food industry actors in producing, marketing, and selling UPF and shaping food environments and behaviors in ways that promote the consumption of these products.^7,8^ To pursue financial growth, the food industry had, in the past and continues to, influence the information on diets and health by engaging and getting closer to health professionals such as dietitians and nutritionists.^9,10^ Industry interactions with nutrition professionals (NPs) could be profitable for NPs, as it could provide extra income and free or reduced rates for continuous education, for instance.^11^ It is also beneficial for the industry to interact and maintain good relations with NPs, as it enhances its corporate image, promotes its products, and creates brand loyalty.^12,13^

 In recent years, however, the interactions between NPs and the industry have raised concerns, particularly their numerous partnerships and sponsorship arrangements, as these may undermine the trustworthiness, integrity, and credibility of the nutrition profession.^14-18^ Concerns have also been raised about health professional influencers – including NPs – receiving industry sponsorship to promote products/services, whether they disclose such funding or not.^15,19,20^ Such concerns about the interactions between industry and NPs have persisted for decades.^21^

 In the medical field, interactions with corporations and their associated risks have garnered much attention and reflection, and have prompted mechanisms to guard against such risks.^22^ More broadly, a scoping review identified four main types of mechanisms for addressing and managing the influence of corporations on public health policy, research and practice (known as corporate political activity): (*a*) transparency; (*b*) management of interactions with industry and conflicts of interest (COIs); (*c*) identification, monitoring and education about the practices of corporations and associated risks to public health; and (*d*) prohibition of interactions with industry.^23^ Recently, work has also been undertaken to develop guidelines for researchers’ interactions with the food industry.^24^ At the individual level, industry interactions with health professionals can create COIs, defined in law and public policy as a situation “where an individual has an obligation to serve a party or perform a role and the individual has either: (1) incentives or (2) conflicting loyalties, which encourage the individual to act in ways that breach his or her obligations” (it should be noted that alternative definitions are used in medicine).^25^ The management of such COI is crucial for maintaining public trust.^26^ Consequently, NPs bodies have re-examined their partnership policies or introduced new guidance.^27,28^ For example, in 2018, Dietitians Australia ended its corporate sponsorship program with organizations within or related to food manufacturing and food industry associations or alcohol companies^29^; they have however been criticized for still allowing advertising by these industries.^30,31^ Moreover, the International Confederation of Dietetic Associations’ International Code of Ethics and Code of Good Practice also explicitly states that NPs should be accountable to the public.^32^

 While studies have been conducted on clinicians’ views of COI/industry interactions,^33-40^ to our knowledge, no review has examined the depth and breadth of interactions between NPs and industry and the perceived benefits, associated risks and solutions. A systematic review of interactions between non-physician clinicians and industry included 15 studies,^41^ only one of which included dietitians in its sample.^42^ As NPs have an important role in improving and maintaining the health of individuals and populations with their activities, it is urgent to examine the interactions between industry and NPs.

 Therefore, this scoping review aimed to map the literature concerning NPs–industry interactions in practice, NPs views or thoughts about those interactions, as well as the risks and solutions to address and manage these risks, and analyse and identify knowledge gaps.

## Methods

 We conducted a scoping review following guidance from Arksey and O’Malley,^43^ Levac et al,^44^ and the Joanna Briggs Institute.^45-47^ The protocol for this study was pre-registered on Open Science Framework (osf.io/2wuda)^48^ and a summary is provided below. The conduct and findings of this scoping review are reported following the Preferred Reporting Items for Systematic reviews and Meta-Analyses Extension for Scoping Reviews (PRISMA-ScR).^49^

###  Stage 1: Identifying the Research Question

 Our general research question was: *What is known from the existing scientific literature about the interactions between NPs and industry?*

####  Sub-questions

- What is the nature of interactions between NPs, at the individual and institutional levels, and the industry, and how extensive are these interactions?
- What are the views of NPs towards those industry interactions, including perceived influence (eg, on professional practice, professional integrity), acceptability, and advantages/disadvantages?
- What are the perceived and observed risks associated with such interactions?
- What strategies/actions have been proposed/used to address and manage those risks?

Our research question, search strategy, and inclusion/exclusion criteria were guided by the PCC (Population, Concept and Context) mnemonic^45^; see Table 1.

**Table 1:** Inclusion and Exclusion Criteria

	**Inclusion Criteria**	**Exclusion Criteria**
Population	Nutrition/dietetic professionals (students/qualified); nutrition/dietetic professional bodies and associations. NPs are “individuals who pursue a professional career in nutrition, such as dietitians or nutritionists, and are trained sufficiently in nutrition practice to demonstrate defined competencies and to meet the certification or registration requirements of national or global nutrition/dietetics professional organizations.”^32^ A professional body is “an organization of people with particular professional qualifications. May seek to set standards of professional competence, to control entry to ensure that its members are able to maintain professional standards to monitor the conduct of members to ensure that they maintain these standards, and to exclude them if they do not.”^50^ National dietetics associations “are professional societies whose members have education qualifications in food, nutrition and dietetics recognized by a national authority and whose members apply the science of nutrition to the feeding and education of groups of people and individuals, in health and disease.”^51^	-
Concept	Industry interactions Industry is defined as companies/corporations that produce food and drink/healthcare goods and services, as well as third parties working for such entities, including their trade associations, public relations firms and associated scientific entities.^41,52^ Pharmaceutical and alcohol industries also, given their presence at nutrition conferences in many countries and their influence on other parts of NPs’ practice.^14^ Interactions are defined as any industry exposure such as meetings with sales representatives; receipt of gifts, payments, or promotional materials, including samples; or attendance at industry-sponsored education.^41^ Risks are defined as unintended, negative consequences of an event for public health, NPs, organization and public policies.^53^	Records focusing on views and experiences of interactions between professionals and industry concerning research as this has been the subject of more recent studies.^54^
Context	Professional practice, not limited by geographic location, language, or year.	Documents solely focusing on NPs working for/in the industry.

Abbreviation: NPs, nutrition professionals.

###  Types of Sources of Evidence

 All scientific study designs were eligible, including those that used qualitative and/or quantitative methods, as well as non-empirical articles, including literature reviews, books, book chapters, guidelines, editorials, opinion pieces, and letters to the editor. Given the exploratory nature of this scoping review, we adopted a non-restrictive approach. We included documents funded by industry or whose authors were employed by industry, but these were analysed separately, given the inherent COI therein.

###  Search Terms

 Title, abstract and keyword searches, using combinations of keywords and Medical Subject Headings (MeSH) (or equivalent), were used across the PCC outlined in Table 1. The search strategy was developed in Medline (documented in protocol^48^), tailored for use within the other databases, and piloted before final searches were run. We developed the research strategy with the help of a librarian at the University of Montreal in Canada.

###  Stage 2: Identifying Relevant Studies

 Between March 17, 2021 and March 21, 2021, VH and MH conducted electronic searches of the following databases/platforms: Scopus (Elsevier), CINAHL Complete (EBSCO host), Embase (OVID), Medline (OVID), CAB Abstracts (CABdirect) and Web of Science Core Collection. We searched for grey literature using Proquest Dissertations and Theses and Google Scholar. We also identified relevant resources through backward and forward citation searching of included records. Records were imported into Covidence software,^55^ where duplicates were automatically identified and removed. We did not seek external expert input to complete the identification of relevant papers that might not have been found through database searches due to the expertise of two members of the review team (MM and JCM).

###  Stage 3: Study Selection

 Title and abstract screening, and subsequent full text reviewing against our eligibility criteria, were conducted by two reviewers (VH and MH); any disagreements were resolved by consensus, or with a third reviewer when necessary (MM). Where full-text was not accessible to the research team, we contacted the author. Two authors were contacted, one did not respond and we finally had access to the other document through the University of Montreal’s library.

###  Stage 4: Charting the Data

 Two reviewers (VH and MH) independently conducted data extraction/charting to reduce the probability of errors and bias^45^; any disagreements were resolved by consensus or with a third reviewer (MM) when necessary. We used a modified version of the Joanna Briggs Institute template to assist with the charting of relevant data, such as author, origin, source type, and results or findings relevant to the review question(s).^45^ Initially, both reviewers independently extracted data from 10% of included records using the data charting table (see protocol^48^). They met to determine whether their approach to data extraction was consistent with the research question and purpose and if it captured the data appropriately.^44,45^ Charting was an iterative process; the form was refined and updated accordingly^45^ – see final data extraction table in Supplementary file 1.

###  Stage 5: Collating, Summarizing, and Reporting the Results

 Following the completion of data charting from included records, we described and analysed the data in two ways. Firstly, we conducted a descriptive numerical summary analysis, encompassing the number and nature of records included in the review. Secondly, VH synthesized the qualitative data extracted for each of the four research sub-questions using content analysis^56^ with an inductive^57,58^ and deductive approach based on previous work about solutions in COI in nutrition^23^ for the sub-question on solutions used or proposed. Data from the results section of the documents, as well as the narrative content of publications, such as commentaries, were analysed qualitatively. We used NVivo software for data management. Finally, a verification of the clarity of the codes was carried out by JCM.^58^ Any disagreements about the codes were discussed and resolved by consensus. Some quotes were categorised under several codes when quotes contained wording relevant to several categories.

## Results

**Figure 1:**
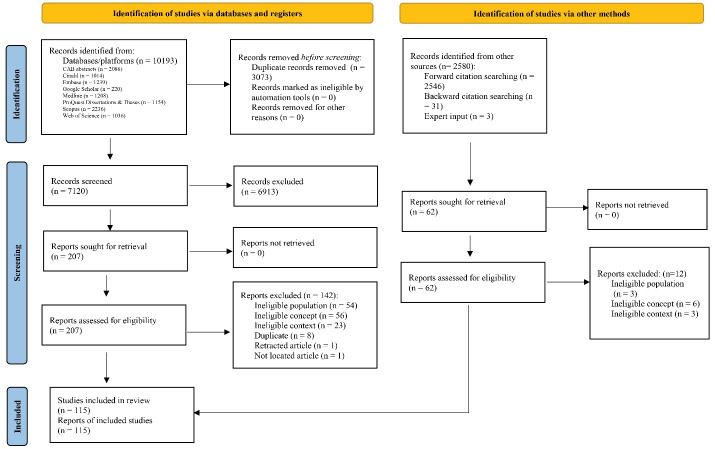
PRISMA Flow Diagram for Our Scoping Review. Abbreviation: PRISMA, Preferred Reporting Items for Systematic reviews and Meta-Analyses

 The PRISMA flow diagram for our scoping review is presented in Figure 1.^59^ In total, 7120 documents were identified through database/platform searches (excluding duplicates) and 2580 via other sources. After title and abstract screening of these 9700 records, and subsequent full-text review of 268 records, we included 115 documents for analysis covering 112 studies (Note: two documents were policy position papers that have been updated/revised; both versions were included^60-63^ — details in Supplementary file 1). Overall, the majority of these documents were identified from original searches (n = 65), while others were obtained from backward citation mapping (n = 23) and forward citation mapping (n = 24), and three additional documents were identified after internal consultation with the team (expert input, Figure 1).

###  Characteristics of Included Documents

 Full details of included documents are provided in Supplementary file 1; key characteristics are outlined in this section.

####  Years

**Figure 2:**
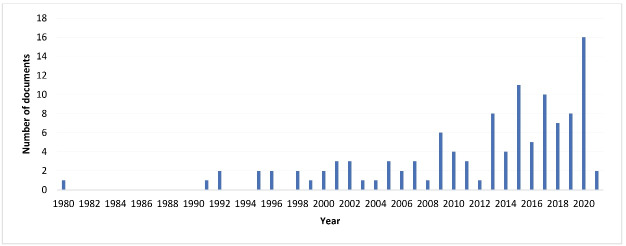
Number of Documents by Year of Publication.

Figure 2 shows the included documents in the scoping review. These were published as early as 1980 with a growing trend, and a majority (90%) were published from 2000 onwards (Figure 2). There is also a significant growth since 2013.

####  Type of Sources 

 The majority of documents were journal articles (n = 91, 79%), including original manuscripts, commentaries, editorials, practice points, policy positions, and letters to editors. Other documents comprised doctoral theses (n = 6, 5%), reports (n = 5, 4%), published conference abstracts (n = 4, 3%), books (n = 3, 3%), book chapters (n = 3, 3%), newsletter articles (n = 1, 1%), and magazine articles (n = 2, 2%).

####  Country of Origin

 Just over half of the documents (n = 59, 51%) were published in/focused on the United States, followed by the United Kingdom (n = 9, 8%), Canada (n = 7, 6%), and Australia (n = 6, 5%). Two documents were from Africa (Nigeria [n = 1, 1%] and South Africa [n = 3, 3%]). Six documents provided global perspectives (n = 6, 5%), while one focused on Europe (n = 1, 1%).

####  Study Design

 Almost two in every three documents were classified as a narrative (n = 63, 55%) or position paper (n = 12, 10%). Only 32% (n = 37) of documents were empirical studies. Almost one in five was a documentary analysis (n = 21, 18%). Other categories of study design included mixed methods (n = 4, 3%), netnography (ie, “qualitative method investigating behaviors of cultures and communities present on the Internet”^64^) (n = 1, 1%), participant observation (n = 1, 1%), qualitative assessment (n = 10, 9%), quantitative and cross-sectional survey (n = 2, 2%) and systematic review (n = 1, 1%) (Supplementary file 2).

####  Context

 Almost a third of the documents focused mostly on individual professional practice (n = 33, 29%). Individual professional practice in nutrition includes professional activities such as individual counselling and/or working in a hospital. ‘Sponsorship of professional body/organization’ was the second most frequent context studied or discussed in the literature (n = 26, 23%), followed by ‘corporate political activity’ (n = 21, 18%) and partnership/collaboration with a professional body (n = 26, 23%) (Table 2).

**Table 2:** Documents by Context and by Population

**Context**	**No. of Articles **	**%**
Professional practice	33	29
Sponsorship of professional body/organization	26	23
Corporate political activity	21	18
Partnership/collaboration with a professional body	21	18
Sponsorship and involvement in scientific events	15	13
Management of COI by professional body/nutrition organization	13	11
NPs education/continuing professional development	10	9
Ethics	5	4
Public nutrition education	3	3
Sponsorship of health professional	2	2
Management of conflict of interest by academia	1	1
Sponsorship/advertising	1	1
**Population **	**No. of Articles **	**%**
Nutrition and health organization	67	59
NPs	59	52
Policy-makers	30	26
Health professionals	28	25
Academics	24	21
Community	13	11
Academia	5	4
Journalists	2	2
Advertisers/publishers	1	1
Opinion leaders	1	1

Abbreviations: NPs, nutrition professionals; COI, conflict of interest. Note: Documents could be categorised under multiple categories; numbers do not add up to 100%. Academia and academics were coded to references to institutions (eg, universities) and individuals, respectively. Nutrition and health organizations were coded for professional bodies, societies, non-profit health organizations and medical associations. Health professionals can include NPs, but these were categorised separately, where possible.

####  Population

Table 2 presents the population studied in the documents analysed. Over half of the documents focused on more than one population (n = 62, 54%). More than half of the documents focused on nutrition and health organizations (including professional bodies, associations and societies) (n = 67, 59%) and NPs (n = 59, 52%).

####  Type of Industry Studied or Discussed 

 Most documents (n = 91, 79%) reported/discussed interactions between NPs and the food and beverage industry, followed by the pharmaceutical industry (n = 29, 25%). Other types of industry reported included the breastmilk substitutes (n = 18, 16%), biotechnology and agrochemical (n = 11, 10%), alcohol (n = 2, 2%) and medical technology (n = 1, 1%) sectors. The majority of documents mentioned more than one type of industry, while some were more general (ie, they did not mention any specific type of industry or company) (n = 15, 13%) (Supplementary file 1).

####  Authors’ Industry Ties

 We examined industry involvement in included documents by analysing the affiliations, declared industry funding sources, and declared COIs. We identified industry ties with authors in 13 documents (11 %). For those papers where industry ties were identified, it was declared through the affiliation(s) of authors^65,66^; in the funding section^67^; the COI section^68,69^; both the affiliation and funding section^70,71^; both in the funding and COI sections^14,21,72^; as well as through the affiliation, funding and declared COI sections.^73-75^ In 24 documents, the information on both COI, funding and affiliations was not stated (it should be noted that the three books included in the review in which COI information is also absent are excluded from that count)^16,17,60-63,76-93^; this included position papers/commentaries from professional bodies known to have had or for having relationships with industry (as declared in documents included or on their website) (ie, Academy of Nutrition and Dietetics [AND],^94^ Society for Nutrition Education and Behavior, and Latin American Society of Nutrition).^28,60,62,63,77,79,80,82,85,89,92,95-98^ Other documents from the AND, the Latin American Society of Nutrition, the British Nutrition Foundation and the Canadian Nutrition Society, who are known to have had or for having relationship(s) with industry (as declared in documents included or on their website^99,100^) have also not declared any industry ties or related COI.^28,101-106^

###  Qualitative Analysis

 We extracted and coded data about types of industry interactions experienced by NPs perceived risks, acceptability, and advantages of industry interactions, as well as strategies and actions used to manage such interactions (see codebooks for details of all codes, with illustrative quotes — Supplementary files 3, 4, and 5). Almost all documents outlined NPs experiences of industry interactions (n = 104, 90%) and perceived risk (n = 86, 75%). More than half of the documents discussed the acceptability and advantages of industry interactions (n = 64, 56% and n = 59, 51%, respectively). Finally, strategies and actions used to manage NPs and industry interactions were outlined in 69% of the documents (n = 79) (Supplementary file 2).

###  Nutrition Professionals’ Experiences of Industry Interactions

 We identified seven channels through which NPs can interact directly or indirectly with industry. These included interactions through (1) NPs, (2) professional bodies or health and nutrition organizations, (3) educational institutions (eg, universities), (4) industry marketing and interactions in work settings, (5) colleagues or other health professionals (eg, physicians), (6) industry website or media advertising and promotional events, and (7) foreign aid context. In total, we identified 32 different categories of interactions between NPs and industry (presented in Table 3). Details of these categories and illustrative quotes can be found in the Supplementary file 3.

**Table 3:** Nutrition Professionals’ Experiences of Industry Interactions According to the Channels Through Which the Industry Interacts With Them

**Channels Through Which the Industry Interacts With NPs **	**Nature of Interactions**
Interactions with individual NPs	Active (solicited or implying involvement from individuals) Employment by or consultation service for industry^21,75,84,96,107-110^ Having stock holding or ownership in an industry^75,89^ Endorsement of a company and its brands ♦ Endorsement of products or industry in the public sphere^89,111-115^ ♦ ndorsement of products in private practice or in the office^78,89,96,107^ ♦ Endorsement and/or co-creation of educational material^90,91,116^ Other direct interactions Receiving travel and conference attendance funding^18,26,42,66,75,91,93,117-119^ Continuing education directly provided by industry^74,83,120-122^ Receiving targeted communication from industry (eg, via letter or email)^111,116,117^
Interaction with professional bodies or nutrition and health organizations	NPs education Involvement in NPs and scientific events ♦ Industry participation in scientific event(s)^14,16,17,21,93,104,110,117-119,121,123-133^ ♦ Exhibit booths^14,21,80,81,90,104,110,115,119,127,128,130-132,134-136^ ♦ Industry promotional and educational materials distribution ^14,21,81,93,115,116,128,130^ ♦ Provision of meals/beverages/food samples^14,21,75,88,93,117,128,130^ ♦ Sponsorship of nutrition events/conferences^9,14,26,78,86,88,93,108,116,118,119,126,128,130,133,135,137-142^ Continuing education provided/sponsored by industry^88,93,103,104,110,135^ Partnership and sponsorship Partnership on programs or advocacy^9,13,16,21,63,67,68,72,73,80,85,90-93,97,103,104,110,115-118,122,126,127,129,131,135,139,143-151^ Sponsorship of professional bodies and health and nutrition organizations^9,16-18,21,76,88,90,91,103,104,110,111,117,118,122,129,131,135,137,138,147,151-157^ Other interactions with professional bodies or nutrition and health organizations Advertising through journal(s), direct mailing, and website^16,21,90,91,93,103,110,111,115,118,129,131,135,137,138,152,158^ Endorsement of food products (such as food certification)^16,26,104,111,115^ Prizes and awards sponsored by or from industry^14,16,26,91,104^ Leaders or committee members with industry's ties or affiliation in the organization^16,90,139^ Networking opportunities (such as membership networking events)^26,74,142^ Industry-led surveys and focus groups with members^13,91^
Interaction with or within educational institutions (eg, universities)	Provision/sponsorship of student educational materials/activities/events/internships^12,71,124,126,132,159^ Scholarships, sponsorship, awards, and prizes for students^12,14,83,126^ Sponsored continuing education^126,143^
Industry marketing/interactions in a work setting	Industry marketing/interactions in a work setting Sales representative visits^42,90,107^ Gifts, samples, and educational material^42,90^ Attending lunchtime meetings (industry representative speaking)^91^ Industry marketing^120^
Interactions through colleagues or other professionals	Indirect influence from industry through colleagues or other professionals (eg, physicians)^78,107,116^
Industry website, media advertising and promotional events	Educational materials and information for professionals and consumers created by the industry and delivered via the web/television advertising or other promotional events (eg, industry had a specific website for health professionals and publishing educational materials for professionals)^110,118,121,122,125^
Foreign aid	Interactions with industry in the context of foreign aid NPs implication^118,160^

Abbreviation: NPs, nutrition professionals.

 When interacting directly with industry, NPs can have ‘active’ or ‘passive’ interactions. More ‘active’ interactions include endorsing industry products for compensation. Examples of active interactions were found in private practices, where NPs received compensation from companies in exchange for recommending their products to clients.^78,89,96,107^ Other direct interactions with the industry that NPs do not necessarily seek out or for which they are not actively involved were also identified, such as receiving targeted communication from the industry (eg, via letter or email)^111,116,117^ (Table 3).

 We identified that professional bodies and health organizations (with whom NPs interact frequently and in many ways) are important channels of influence for the industry through various interactions (Table 3). The most commonly reported interaction was industry participation in NPs continuing education within professional bodies and health and nutrition organizations. For example, 22 documents attested that the industry participated in various nutrition and scientific events around the world by having industry-sponsored or affiliated speakers and holding specific sessions, conferences and symposia.^14,16,17,21,93,104,110,117-119,121,123-133^ Other examples include industry involvement in seminars and training (continuing education) provided by professional bodies.^88,93,103,104,110,135^

 Partnerships between professional bodies or health and nutrition organizations and industry were reported in 39 documents, while sponsorship of these organizations was reported in 29 documents (Table 3). Sponsorship of the AND was mentioned in 18 of these documents,^9,21,76,88,90,93,104,110,117,118,129,131,138,147,151,153,156,157^ followed by the British Dietetic Association (BDA) (n = 3) ^18,91,152^ and the Dietitian Association of Australia (DAA) (n = 3).^16,111,152^ It was also reported in Canada,^155^ New Zealand,^103^ Spain,^135,145^ and South Africa.^122^ Professional bodies and nutrition and health organizations also gave industry access to NPs through their actions, communications, and structures, by, for instance, advertising in journals, on the organization’s website and by direct mailing.^16,21,90,91,93,103,110,111,115,118,129,131,135,137,138,152,158^

 We identified three types of interaction that occurred within educational institutions, such as the provision or sponsorship of nutrition student educational materials, activities, events and internships^12,71,124,126,132,159^ (Table 3). Other channels through which the industry interacts with NPs are NPs workplace, NPs colleagues or other health professionals and industry websites and media advertising and promotional events. Finally, interactions with industry were also identified in the context of foreign aid.^118,160^

###  Nutrition Professional’s Acceptability of Industry Interactions 

 Acceptability of industry interactions varied widely. Some authors, NPs (surveyed or interviewed), and organizations perceived interactions as acceptable,^9,14,42,62,77,88,90,91^, ^93,104,116-118,128,130-132,138,142,157,158^ or even encouraged them.^21,60,61,63,65,73,76,79,80,90-92,95,97,98,101-104,116,131,132,142,161,162^ For those who actively encouraged these interactions, industry was considered an ally in promoting public health and developing such relationships. It is important to note that commentaries, position papers and letters of presidents from nutrition organizations and professional bodies (ie, AND, Canadian Nutrition Society and the British Nutrition Foundation) all encouraged interactions with industry,^60,61,65,79,80,92,95,97,98,101-104^ or considered these as being acceptable.^62,77,104^ Nevertheless, this review and further searches revealed that these organizations have several ties with industry, which could explain their stance.^94,99,100^

 Some considered that acceptability was conditional on the type of industry.^79,81,91,93,110,116,138^ For instance, some authors or NPs stated that industry’s mission should be consistent with their organization.^79,138^ The nutritional values or level of processing of companies’ products were other conditions influencing acceptability. In a survey conducted with AND members,^138^ “food growers and producers” were perceived the most acceptable to work with, while “food and drink manufacturers” were deemed the least acceptable. Acceptability could also be conditional on the type of interaction (eg, free travel and accommodation were acceptable, but involvement in nutrition/scientific events were not),^9,21,90,91,118^ or on other conditions such as following a code of ethics or being transparent.^75,77,83,87,89,91,96,105,115,132,142^ On the 11 documents that welcomed interactions with industry if a code of ethics is followed or if those are undertaken with transparency, five were documents from the AND.^75,77,89,96,105^

 Finally, the perception that interactions with industry were not acceptable was reported in 22 documents.^14,16,18,78,85,88,90,91,93,106,110,116,118,128,131,132,138,140,151,155,157,160^ For instance, Bellatti^151^ reported that some members renounced their AND memberships because of its history of ties with the food industry, which demonstrates a high level of unacceptability. It may be noted that of the documents presenting an unfavourable stance about interactions with industry, none appear to have COI or indirect ties with industry through nutrition organizations like AND.

###  Perceived Advantages of Industry Interactions 

 A variety of advantages associated with industry interactions were perceived by some authors and NPs.

####  Advantages for Organizations

 Interacting with the industry provided three benefits for organizations. First, interactions were perceived as beneficial for the financial survival of organizations.^16,26,63,77,80,88,90,93,104,111,115,130,138,155,157^ Second, others highlighted that sponsorship and funding were a way to earn additional income and accelerate business growth. For example, extra income could allow organizations to hold scientific events in a prestigious location or to offer more activities within those events.^104,118,138,142^ Third, it allowed organizations to fulfil their mission to a greater extent (eg, by having facilitated/funded educational programs or other activities that could not otherwise have happened).^62,63,76,92,104,142^ In 1995, a past president of the AND (formerly called American Dietetic Association) highlighted that “[i]ndustry support makes an invaluable contribution toward fulfilling the mission and vision of American Dietetic Association, and can help accomplish many activities at the local level.”^63^ Twenty years later, in 2015, NPs from the AND still endorsed this view.^104^

####  Advantages for Professionals and Organizations

 Advantages of industry interactions that apply to both professional bodies/organizations and professionals included improving public outreach,^62,63,80,104,115,116,131,157^ benefiting from industry’s expertise (eg, marketing and public relations expertise, skills and networks),^16,63,88,104,142^ building awareness of the professional body and its members to the public,^80,103,104,157^ and enhancing credibility and reputation.^63,104^

####  Advantages for Individual Professionals

 Continuing education and information,^42,71,74,78,88,97,98,104,115,131,157^ career and employment opportunities,^66,80,88,104,107,118^ source of income^9,85,89,111-113,115^ and prestige^96,111^ were reported benefits that professionals could personally gain from industry interactions.

####  Advantages for the Public

 Some benefits that the public could gain from the interactions between industry and NPs were described. Indeed, it was argued that those interactions could positively influence industry actions and product development toward more healthy food products.^9,16,79,88,95,101,102,104,115,131,142,148,157^ For others, these interactions could shape public food choices and improve public health (eg, by combining resources,^157^ “singles out products useful from standpoint of professionals”^115^ or “promot[ing] environments and messages that facilitate healthy food […] choices”^98^),^16,61-63,73,86,98,104,115,129,131,145,163^ as well as offer better population nutrition education and information through partnerships (eg, by providing public with good nutrition materials at no charge).^16,60,61,63,65,92,101,103,104,115,116^

###  Perceived and Experienced Risks of Interacting With Industry

 Several risks were identified across the documents encompassing general risks and some specific to professionals, organizations, and the public.

####  General 

 According to the literature, relationships between NPs and industry actors could result in the public, NPs or their professional body refraining from criticizing industry actions or from encouraging people to engage in critical thinking around industry behavior and actions.^9,16,21,81,93,110,111,117,118,122,128,132,143,146,150,155^ Interactions also represent a risk of being a vehicle for industry marketing and messaging^14,16,17,21,41,93,109,116,130-133,135,138,146,157^ and creating positive associations and credibility for industry brand(s).^12,13,93,106,108,110,128,135,136,139,141,149,155^

####  For Health Policy

 Interactions between NPs and industry can also influence public health policies, given that professional bodies and nutrition and health organizations are respected and influential in their countries. For example, AND has a political action committee. Some NPs formally advise governments and advocate for nutrition policies.^93,164^ Many authors pointed out that interactions could contribute to framing the debate around food and health in a way that could be favourable to the industry in two ways. First, by influencing NPs and professional bodies on food products and public health messages through industry-friendly narratives (eg, there are no good or bad foods, favouring energy balance and moderation, and focusing on individual choices)^16,21,28,68,88,116,128,129,132,146,147,151,157,162,165^ and second, shaping policy positions of professional bodies and health organizations.^16,17,93,118,137^ For instance, corporate sponsorship has shaped the policy positions of nutrition organizations, such as the Spanish Federation of Nutrition, Food and Dietetics Societies, which opposed the Nutri-Score front-of-pack labelling system, otherwise supported by health organizations across Europe. Interactions could also introduce bias in policies or dietary guidelines and programs^76,127,128,139,141^ and favour corporation lobby efforts aimed at delaying or neutralizing public health policies such as soda taxes or dietary guidelines (eg, by “invoking reciprocity and financial dependence on the part of national health organizations”^156^).^13,139^

####  For Professionals and Organizations

 Risks of interacting with industry that apply to both organizations and professionals included image and reputational risks, including undermining trust, credibility, integrity and reputation^14,16,17,21,26,28,65,75,78,79,81,84,86,88,90,91,93,96,104,108,110,115,118,128,131,142,144,153,155,157,166,167^ and appearance of endorsement of brands or products or commercial bias.^21,77,90,91,115,117,138,143^ It can also compromise independence by impairing objectivity and judgement^9,14,21,26,68,75,83,85,91,96,115,116,130,137,143,152,153,155,167^ and influencing decision-making or recommendations.^12,13,41,75,77,90,93,96,105,106,111,115,128,137,158,165,167^ Finally, interactions with industry can make the organization lose members^151^ and even contradict the organization’s and professional’s public health mission.^93,110,115,141,157^

####  For Professionals

 For individual NPs, interactions with industry could pose several risks, including the influence on the scientific content of nutrition events (eg, cancelling a debate on childhood obesity because it would cause inconvenience to potential sponsors^130^ or providing educational flyers with commercial bias^128^).^93,110,137,140,151^ It can also influence the content of continuing education programs^88,93,110,167^ and students’ training/teaching programs and careers.^12^ This influence can be translated into a risk of commercial bias and incomplete education, as described by Simon: “Equally concerning, if registered dietitians are getting their continuing education units from the food industry, what messages are they missing? Coca-Cola or General Mills are not going to sponsor sessions on the harmful impacts of marketing to children despite the numerous studies demonstrating the connection.”^93^ NPs’ practices and beliefs may also be unconsciously influenced.^110,111,116,131,155,156^ This risk was illustrated in the context of collaboration between NPs and the food industry in school nutrition programs: “[…] these relationships leave NPs open to the charge that their lack of attention to food industry marketing efforts in elementary schools arises from their close ties to the food industry.”^116^

 Two documents also reported that industry interactions with NPs and health and nutrition organizations could be misleading and result in confusion for NPs (eg, by confusing sponsorship and health promotion).^111,118^ Less frequently reported were risks of creating antagonism between health professionals^108^ and the revocation of licensure in case of ethical issues.^89^

####  For the Public

 NPs are respected professionals, capable of influencing population knowledge and perceptions about nutrition.^168^ Our review suggests that interactions with industry may impact the public in three ways. First, interactions may mislead and confuse the public about nutrition knowledge (eg, by confusing nutritional advice with sponsor’s marketing or by not receiving all the information about foods).^26,66,78,84,88,90,91,108,110,115,117,118,120,153,157^ Second, they could undermine public health nutrition recommendations,^13,18,42,76,84,117,118,130,133,135,137,139,141,143,144,146,157,158,160,165^ as illustrated by Potvin-Kent and al: “[…] [P]artnering with food companies, particularly those that largely produce and promote unhealthy food products, could confer an aura of healthfulness, goodwill and credibility to these industry partners while eclipsing the fact that many of the same companies or their industry associations persistently and aggressively push-back against government policies and the efforts of public health advocates aimed at improving diet and health.”^144^ Third, one document reported that “price of product may be increased (or not so low as it could be) due to costs of endorsement.”^115^

###  Strategies and Actions, Proposed or Used to Address and Manage the Risks Associated With NPs and Industry Interactions

 We classified strategies and actions, proposed, used, or in use, to address and manage the risks associated with interactions between industry and NPs, according to whom these strategies or actions apply, either at the institution, individual or both levels. Five main categories of strategies were identified, namely (1) management, (2) education, (3) prohibition, (4) transparency, and disclosure (5) awareness-raising. Table 4 presents all these strategies. Further details on these strategies and illustrative quotations can be found in Supplementary file 5.

**Table 4:** Strategies and Actions, Proposed or Used to Address and Manage the Risks Associated With Nutrition Professionals and Industry Interactions

**Type of Strategy**	**Sub-categories of Strategy**
**Institution/Organization/Professional Body Level**	
Management	1.1) Codes, policies, and guidelines^9,12,16,18,21,26,28,41,42,75,79,81,82,85,88,90,93,96,106,109,113,115,122,125,131,135,138,143,152,155,157,165^ Monitoring and evaluation of its respect^42,87,142,143^ Sanction when non-compliance observed^14^ Having or developing more selective criteria for choosing sponsors^63,93,104,130,138,141,157^ Dissemination of codes and guidelines to mitigate or manage COI^26,79,106,157^ Invest in human resources to assist with COI management^88^ 1.2) External and internal consultation Consider members’ opinions regarding institutional sponsorship^88,91,118,138,157^ Creating a COI/ethics committee^12,81,82,106,128,130,133^ Independent advisory group to assist with COI management^88^ 1.3) Alternative financial strategies Seek alternative/non-conflicted sponsorship or funding^16,28,88,118,130,142,144,157^ Cut down expenses and revise priorities^14,81,88,118,126,142,157^ Increase membership fee revenue^81,138^ 1.4) Compromise and other strategies Structural changes at conferences/events^78,101,115,125^ Accept less risky interactions^9,91,143^ Ensure the educational materials used are free of explicit or implicit bias^115^ Dilution principle^91,138^
Education	1.5) Educate NPs about industry interactions and related COI^12,41,66,81,88,107,131,132,143,155^ 1.6)Strategies to favor independence in educational settings Independent accreditation of university dietetic training^16^ Use independent documentation, references, and teaching materials^90^ Present a variety of products instead of a particular brand^90^
**Individual Level**	
Management	2.1) Tools or resources for decision-making Code of ethics and guidelines^18,21,66,75,77,84,87,89,90,96,103,107,112,114,157,165^ Other tools (DORM and Nolan principles)^78,86^ 2.2) Individual discernment Balance risks and benefits on a case-by-case basis^9,21,75,144^ Fact and references checking and follow up^16,90,131^ Rely on professional judgment^77,131^ 2.3) External consultation and advice on COI^77,78,96,106,132^ 2.4) Accepting funding through third-party^117^ 2.5) Retain control over content^75^ 2.6) Document COI management^78^
**Institution/Organization/Professional Body and Individual Level**	
Prohibition	3.1) Prohibiting, avoiding, and refusing all interactions with industry
Transparency	3.2) Transparency and disclosure Transparency of institution vis-à-vis the public and members^9,26,87,91,122,127,137,139,157^ Transparency in conferences and nutrition and scientific events^75,90,96,104,105,118,128,130,142,165^ Signed agreement or contract with industry to manage/guide interactions around conference sponsorship or general sponsorship^16,118,137^ Transparency of NPs vis-à-vis the clients and the public^21,66,75,78,89,96,109,112,114,117,143^
Awareness-raising	3.3) Identification and awareness-raising Advocating for COI recognition and action by institutions^12,81,106,118^ Identifying COI^21,78,117^

Abbreviations: NPs, nutrition professionals; COI, conflict of interest; DORM, Disclosure, Options, Reassurance, Modification.

###  Institution/Organization/Professional Body Level 

####  Management 

 Institutions, organizations and professional bodies use various management strategies to handle industry interactions. These include (1) codes, policies, and guidelines, (2) external and internal consultation, (3) alternative financial strategies, (4) compromise, and (5) other strategies (Table 4). The most commonly used strategy is to follow and develop codes, policies, and guidelines (and revise these if they are not considered strong enough or adequate). However, implementing these can be problematic, as some organizations have been found to deviate from their own established rules. Indeed, it was reported that the DAA, AND, and the BDA undertook activities that conflicted with or deviated from their own established code, guidelines, or policies.^16,91,93^ For instance, Simon identified that “[…] [t]he DAA’s policy on brand endorsement is contradicted numerous times, for example, on the DAA’s Pinterest pages, with recipes credited to companies such as Unilever, Campbell’s, and Nestlé that list branded products as ingredients.”^16^

 Otherwise, some authors suggest more selective criteria for choosing sponsors in industry partnerships with organizations.^130,138,141,157^ For instance, it was reported that the AND already had a list of “General Requirements for Acceptance of Corporate Relations Sponsors.” The list however did not appear to be used by the organization.^93^ Moreover, whereas NPs surveyed by Reitshamer and colleagues in 2012 mentioned that AND must be more selective in choosing sponsors,^138^ two other documents from the AND mentioned that the organization was already choosing their sponsors and partners based on “well-defined criteria” and “complex and rigorous scrutiny.”^63,104^

 Other strategies proposed included structural changes at conferences/events^81,104,118,128^ (eg, having a commercial area separate from the scientific content) and including additional sponsors to reduce specific industry influence (dilution principle).^91,138^

####  Education

 Educating professionals about the issue was another strategy proposed or used.^12,41,66,81,88,107,131,132,143,155^ Some suggested that NPs should be “invited to discuss the moral and ethical implications of doing business with a variety of private food and pharmaceutical corporations”^155^ and these “ethical implications should be problematized […] during continuing education.”^41^ In four documents, it was suggested that the issue should be included in courses and in projects within educational institutions where NPs are trained.^12,131,132,143^

###  Individual Level

####  Management

 We identified six main individual-level management strategies (Table 4). The most commonly suggested strategy was using tools like a code of ethics to manage COI when making decisions.^18,21,66,75,77,78,84,86,87,89,90,96,103,107,112,114,157,165^ Balancing risks and benefits on a case-by-case basis (eg, in the context of corporate funding^9,21,144^ or accepting gift or payment^75^) is an example of the “individual discernment” strategy proposed. Another strategy proposed by an author with industry ties was that “[NPs] can ethically act as consultants and speak on behalf of a company or product as long as they retain control over the content and disclose their relationship with the company.”^75^

###  Both Institution and Individual Level Strategy

####  Prohibition: Prohibiting, Avoiding, and Refusing Interactions With Industry 

 Some authors proposed prohibition as a strategy to deal with interactions with industry, including avoiding, refusing, and prohibiting all interactions. Some suggested refusing invitations and gifts from industry at the individual level (eg, declining invitations to attend or speak at sponsored meetings),^9,75,77,78,106^ while others mentioned avoiding certain situations (eg, avoiding visiting industry booths at nutrition events or not attending a presentation that indirectly endorses certain products).^89,93,118,131^

####  Transparency: Disclosing Interactions With Industry and Related COI

 Transparency was proposed as a strategy for organizations and individuals to mitigate interactions with industry (Table 4). Nine documents highlighted the importance of transparency for institutions to be open with the public and members about their interactions with industry.^9,26,87,91,122,127,137,139,157^ While these authors and NPs called for more transparency from some organizations such as the AND and the BDA, three documents from the AND emphasized ongoing efforts around transparency.^28,79,96^

 However, despite transparency being heavily discussed in documents where authors have industry ties (directly or through AND and the American Society of Nutrition),^26,28,66,75,79,89,96,104,105^ only one has declared their industry affiliation.^66^ A lack of transparency from organizers of nutrition and scientific events about industry ties has also been identified in some documents.^14,16,17,93^ For instance, Mialon et al found that many conferences in Latin America and the Caribbean in 2018-2019 lacked information about food industry involvement.^14^

####  Awareness-Raising: Advocating for COI Recognition and Action to Address COI by Institution

 Another reported strategy was for members of professional bodies and organizations to advocate for recognition of COI resulting from industry interactions.^12,81,106,118^ Two advocacy groups identified were Dietitians for Professional Integrity and a group of researchers and NPs who were members of the Latin American Society of Nutrition. They have both started a petition to ask for their organization to recognize COI and take necessary action. Finally, it was suggested that professionals and their organizations reflect on their existing interactions with industry to identify COI.^21,78,117^ One author further proposed a framework to help with this work.^78^

## Discussion

 This scoping review aimed to map the literature on NPs–industry interactions in practice, as well as professionals’ views about the acceptability, advantages and risks of those interactions and the solutions to address and manage these risks. We identified numerous categories of interactions (n = 32) that can occur between NPs and different types of industry. Interactions were primarily with food and beverages industries, but other industries also interacted with NPs (eg, pharmaceutical and breastmilk substitutes). This review also highlights the need to consider various settings and points of influence in the career paths of NPs (eg, initial training, workplaces, etc) which can increase the risks identified in this review and discussed below. Some of these interactions, such as industry representatives visits, meals, product samples and gifts distribution, educational events and educational materials distribution, and payment for travel and accommodation attendance are not unique to NPs. These interactions have also been identified and discussed in other health sectors such as nursing, doctors of pharmacy, physiotherapists, and physicians.^22,41,109,169^

 We found that the acceptability of those interactions varied considerably among authors and NPs. While some authors and NPs encouraged or considered interactions with industry acceptable, others were more nuanced or considered them unacceptable. This review also revealed that documents from associations and professional bodies that had ties with industry tended to encourage and consider interactions with industry as being acceptable. On the other side, from all the documents revealing stances not favourable to these interactions, none have declared COI. Moreover, surveys^91,116,138^ and interviews^88,131,157^ from documents included in this review also showed that acceptability varied through members of the same association, such as AND and BDA. This variability is also reflected across other health professions. Indeed, in a systematic review of interactions between industry and other professionals than physicians (including NPs), Grundy^41^ identified that a majority of professionals held favourable views of industry interactions (such as sale representatives visits), while only a minority held negative views toward such interactions.

 We identified 14 advantages of interactions with industry perceived by different authors and NPs in the documents. Financial support was the most common benefit, aiding both organizations (financial survival, additional income and business growth) and NPs (source of income and career/employment). Similarly, Grundy and colleagues also reported that nurses believed it would be impossible to do their jobs without industry resources.^169^ Industry expertise is another advantage identified in our review. However, expertise transfer from industry is problematic because this expertise is oriented toward profit creation, marketing and brand loyalty.^170^ Despite the benefits, it is important to note that risks also exist and can outweigh these advantages.

 Our review found risks associated with interactions between NPs, organizations, the public, and public policies. One frequently cited risk was the potential damage to the image and reputations of NPs and their professional organizations, which is also recognized in other professions.^22,170,171^ These interactions also posed a risk for public health policies by introducing bias in public policies and programs and potentially favouring industry lobbying. These actions are part of a larger set of corporate political activity strategies that aim to influence policy in ways that benefit industry profitability at the expense of public health.^108,121,125,127,172-177^ These strategies have been previously identified in tobacco research, which undermined and delayed public health policies aiming at controlling product sales, use and distribution.^129^ Although there is limited evidence of the actual effects of the interactions described here at the individual level, similar interactions in medicine have been shown to impact the behaviour and quality of prescription of medical doctors who engage with industry representatives.^178^

 Many strategies were proposed or used to manage and address risks associated with interactions between NPs and industry. Transparency was the most frequently mentioned strategy for both individuals and institutions. However, this strategy alone might not be enough to mitigate the risks and ensure trustworthiness, indeed, it can also “guild the lily” even more.^170,179^ Another questionable strategy identified in our review is the “dilution” strategy, which consists of having multiple partnerships or sponsors to reduce the influence of any single corporation. It is argued that this approach may exacerbate the framing effects by having a cumulative effect of influence, instead of reducing or diluting it.^170^

 We did not assess the adequacy of the proposed solutions because it was beyond the scope of our review. However, as discussed above, some of these solutions are questionable. Notably, some of those were proposed by authors that had themselves COI or ties with industry, such as the author Woteki,^75^ who proposed managing interactions by retaining control over the content, and the documents from the AND who promoted transparency. Moreover, simply having a code of ethics and guidelines may not be enough to protect the profession, as implementation can be problematic. As mentioned above, some institutions deviated from their established code, guidelines, or policies and/or lacked transparency.^14,16,17,91,93^ To address this, recommendations include revising, evaluating and monitoring the respect of codes, guidelines and policies^42,87,142,143^ and applying sanctions for non-compliance.^14^

 Evidence of the most effective strategies to mitigate COI and risks associated with interactions with industry within public health is still limited. One promising strategy to consider is prohibition, based on the effect it has had on tobacco control. Under the 5.3 Article of the World Health Organization (WHO) Framework Convention on Tobacco Control, which is adopted in national Law in 182 countries across the globe,^180^ any individual working in the public sector and involved in tobacco control policies cannot interact with the tobacco industry.^181^ Some argued that national initiatives, ensuring independence and transparency of policy-making, such as the implementation of article 5.3 of the Framework by countries, have been effective.^181^ Thus, it has been suggested that this kind of initiative could be replicated for other industries which negatively impact health, such as the UPFs industry.^181^

 Education, another proposed solution, can be a first important step toward better independence, with lessons to draw from the medical field and pharmaceutical industry influence. One example of this type of strategy is the development of educational materials for medical students and practitioners made by the WHO and the Health Action International, *Understanding and Responding to Pharmaceutical Promotion - A Practical Guide, *releasedin 2013.^22^ More recently, the research team called “PEPITe santé” in France developed a training for critical analysis of pharmaceutical promotion for medical students.^182-184^ These training programs could be adapted for NPs since many interactions and risks identified in this scoping were similar to those identified in this area.^22^ Raising NPs awareness of the various interactions with industry and the risks attached is necessary and should be included in all dietetic programmes and continuous professional training.

 Some promising movements to counter inappropriate sponsorship of nutrition and health organizations had emerged in the past decade. In 2013, a grassroots organization, Dietitians for Professional Integrity, was formed to advocate for the AND to sever its ties with food industry partners and sponsors,^81^ though the organization disbanded five years later as it failed to achieve its objectives. The Hunger and Environmental Nutrition Dietetic Practice Group of the AND has also publicly criticized the Academy’s sponsorship practices.^157^ Although this advocacy resulted in toolkits for non-members and members^185^ and guidance to help AND better choose sponsors,^185^ the organization is still supported by corporations manufacturing UPF, such as Mondelēz International.

###  Implications for Research

 This review found that NPs-industry interactions are gaining attention in the literature, but empirical studies are limited and mainly focus on the United States. More research is needed to systematically document industry interactions with NPs and the impacts and risks associated with these. Research on strategies to manage NPs-industry interactions and COI is needed as fewer studies have focused on this area.^23^ Future research in the area should focus on media, particularly social media (including blogs), given the rise in their prominence^186^ and also examine industry documents to gain insights on this issue from an industry perspective.

###  Strengths and Limitations 

 This study has several strengths. Firstly, our scoping review presents a comprehensive overview of the literature on NPs interactions with the industry. A further strength of the study is the synthesis and reporting of the qualitative data from the scoping review, which goes beyond the traditional scope of a review. This provides valuable evidence on which to base future research and inform practice.

 We did not assess the quality of the included documents. However, this was not our focus or within the remit of a scoping review per se, as we set out to map the literature in this area to inform future research. Lastly, it is important to note that we captured some hypothetical situations in our analysis which illustrated existing types of interactions between NPs and industry, ie, authors did not provide actual examples and/or citations.^66,75,77,78,84,86,90,96^

###  Deviations From Protocol

 First, we planned to search professional bodies’ websites, selecting the most relevant ones based on the initial findings from the previous searches. However, we did not proceed with this approach due to the high volume of records identified. We also initially planned to extract data regarding the ‘disadvantages of industry interactions’; however, on piloting, there was an overlap between this and ‘perceived risks,’ so we merged it into the latter column. There was also an overlap between the ‘Views of NPs towards the perceived influence of industry interactions’ and advantages and risks; we amended the former to general views.

## Conclusion

 NPs have a crucial role in identifying and addressing inappropriate commercial practices, while promoting nutrition for health.^187^ Our scoping review identified several areas for future research, such as exploring the impact of these interactions on nutrition practice and public health policies. Finally, to better manage the COI resulting from these interactions, reviewing and monitoring existing institutional policies and guidelines and evaluating the effectiveness of current solutions through research could be first steps to enhance transparency, accountability, and ultimately the quality of nutrition care.

## Acknowledgements

 We wish to thank Myrian Grondin, Librarian at the University of Montreal, for her assistance in the development of the search strategy.

## Ethical issues

 Not applicable.

## Competing interests

 Authors declare that they have no competing interests.

## Funding

 There was no dedicated funding for this review. VH received financial support from the Centre de Recherche en Santé Publique (CReSP) when this study was conducted and is financially supported by the Fonds de Recherche du Québec – Santé [Grant Number: BF2 – 319234]. MH was a postdoctoral researcher funded by the Health Research Board-Ireland [Grant Number: ILP-HSR-2019-011] when this study was conducted. MM is funded by the Health Research Board-Ireland [Grant Number: ARPP-2020-002]. JCM is funded by the Canadian Institutes of Health Research, the International Development Research Center (IDRC) and Heart & Stroke Canada [Grant Numbers: RNI00488, RY000380, RQ000690].

## Supplementary files


Supplementary file 1. Data Extraction Table.

Supplementary file 2. Overview of Included Documents in This Scoping Review.

Supplementary file 3. Codebook With Illustrative Quotes – Nutrition Professionals Experiences of Industry Interactions and Acceptability.

Supplementary file 4. Codebook With Illustrative Quotes – Perceived Advantages and Risks.

Supplementary file 5. Codebook With Illustrative Quotes – Strategies and Actions Proposed or Used, to Address and Manage the Risks Associated With Nutrition Professionals and Industry Interactions.
