# Supplementary material for: Interactions Between Nutrition Professionals and Industry: A Scoping Review
Source: Int J Health Policy Manag. 2023 Aug 22;12:7626. doi: 10.34172/ijhpm.2023.7626 (PMC10590255; doi:10.34172/ijhpm.2023.7626)
Supplement: Supplementary file 2 — Overview of Included Documents in This Scoping Review. [file ijhpm-12-7626-s002.pdf]

Article title: Interactions Between Nutrition Professionals and Industry: A Scoping Review

Journal name: International Journal of Health Policy and Management (IJHPM)

Authors' information: Virginie Hamel[1,2]*, Marita Hennessy[3], Mélissa Mialon[4], Jean-Claude Moubarac[1,2]

[1]Department of Nutrition, Faculty of Medicine, University of Montreal, Montreal, QC, Canada.

[2]Centre de recherche en Santé publique, Montreal, QC, Canada.

[3]College of Medicine and Health, University College Cork, Cork, Ireland.

[4]Trinity Business School, Trinity College Dublin, Dublin, Ireland.

***Correspondence to**: Virginie Hamel, Email: virginie.hamel.1@umontreal.ca

Citation: Hamel V, Hennessy M, Mialon M, Moubarac JC. Interactions between nutrition professionals and industry: a scoping review. Int J Health Policy Manag. 2023;12:7626. doi:10.34172/ijhpm.2023.7626

Supplementary file 2. Overview of Included Documents in This Scoping Review

Author, year	Origin /Country of origin (where the source was conducted /published)	Experiences	Risks	Acceptability	Advantages	Strategies	Empirical data
Aaron, 2017	United States	Yes	Yes	Not stated	Not stated	Yes	Yes
Aksnes, 2017	United States	Yes	Yes	Yes	Yes	Yes	No
Ashley, 1995	United States	Not stated	Not stated	Yes	Yes	Not stated	No
Ayoob, 2002	United States	Not stated	Not stated	Yes	Yes	Not stated	No
Baker, 2021	Global	Yes	Yes	Yes	Not stated	Yes	Yes
Barlow, 2018	Global	Yes	Not stated	Not stated	Not stated	Not stated	Yes

**Author, year**	**Origin /Country of origin (where the source was conducted /published)**	**Experiences**	**Risks**	**Acceptability**	**Advantages**	**Strategies**	**Empirical data**
Barquera, 2018	Latin America (with authors from Mexico, Chile, Guatemala, & the US)	Yes	Yes	Yes	Not stated	Yes	No
Barquera, 2020	Mexico	Yes	Yes	Not stated	Yes	Yes	No
Bellatti, 2019	United States	Yes	Yes	Yes	Not stated	Yes	No
Bergman, 2010	United States	Yes	Not stated	Yes	Yes	Not stated	No
Berning, 2007	United States	Not stated	Yes	Not stated	Not stated	Yes	No
Boyce, 2017	United States	Yes	Yes	Yes	Yes	Yes	No
Briggs, 2010	United States	Not stated	Not stated	Yes	Yes	Not stated	No
Brownell, 2009	United States	Yes	Yes	Not stated	Yes	Yes	No
Bruhn, 2006	United States	Not stated	Yes	Yes	Not stated	Yes	No
Calder, 2020	Global	Not stated	Yes	Not stated	Not stated	Yes	No
Canella, 2015	Brazil	Yes	Yes	Not stated	Yes	Yes	No
Chan, 2020	United States	Yes	Not stated	Not stated	Yes	Yes	Yes

Author, year	Origin /Country of origin (where the source was conducted /published)	Experiences	Risks	Acceptability	Advantages	Strategies	Empirical data
Clapp, 2017	Global (Canada, Australia)	Yes	Not stated	Not stated	Not stated	Not stated	No
Cohen, 2009	Canada	Yes	Yes	Yes	Yes	Not stated	No
Connor, 2015	United States	Yes	Yes	Yes	Yes	Yes	No
Crowther, 1999	Indonesia	Yes	Not stated	Not stated	Yes	Not stated	No
Derelian, 1995	United States	Yes	Not stated	Yes	Yes	Yes	No
Derelian, 1996	United States	Yes	Not stated	Yes	Yes	Yes	No
Diekman, 2007	United States	Yes	Not stated	Yes	Yes	Yes	No
Dietitians for professional integrity, 2013	United States	Yes	Yes	Yes	Not stated	Yes	No
DiMaria-Ghalili, 2014	United States	Yes	Not stated	Not stated	Yes	Not stated	No
Dixon, 2004	Australia	Yes	Yes	Yes	Yes	Not stated	Yes
Dumbili, 2019	Nigeria	Yes	Yes	Not stated	Not stated	Yes	Yes
Ernst, 1991	United Kingdom	Not stated	Not stated	Yes	Yes	Not stated	No
Finn, 2005	United States	Yes	Yes	Yes	Yes	Not stated	No
Flint, 2015	United Kingdom	Yes	Yes	Yes	Not stated	Yes	No

**Author, year**	**Origin /Country of origin (where the source was conducted /published)**	**Experiences**	**Risks**	**Acceptability**	**Advantages**	**Strategies**	**Empirical data**
Flint, 2016	United Kingdom	Yes	Yes	Yes	Not stated	Yes	No
Fornari, 2001	United States	Yes	Yes	Not stated	Yes	Yes	Yes
Freedhoff , 2011	Canada	Yes	Yes	Not stated	Not stated	Yes	No
Freedhoff, 2014	Canada	Yes	Yes	Not stated	Not stated	Yes	No
Freeland-Graves, 2013	United States	Yes	Not stated	Yes	Yes	Not stated	No
Freeland-Graves, 2002	United States	Yes	Yes	Yes	Yes	Not stated	No
GarciaChavez, 2017	Latin America [Mexico]	Yes	Yes	Yes	Not stated	Yes	No
Garza, 2019	United States	Yes	Yes	Not stated	Yes	Yes	No
Gingras, 2005	Canada	Yes	Yes	Yes	Yes	Yes	No
Gomes, 2013	Global / Spain (Granada)	Yes	Yes	Yes	Yes	Yes	No
Gramlich, 2011	Canada	Not stated	Yes	Yes	Yes	Not stated	No
Grundy , 2013	United States	Yes	Yes	Yes	Yes	Yes	Yes
Gussow, 1980	United States	Yes	Yes	Yes	Not stated	Not stated	No

Author, year	Origin /Country of origin (where the source was conducted /published)	Experiences	Risks	Acceptability	Advantages	Strategies	Empirical data
Guzman-Caro, 2020	Spain	Yes	Yes	Not stated	Not stated	Yes	Yes
Helm, 2016	United States	Yes	Yes	Not stated	Yes	Yes	No
Hermann, 1992	United States	Yes	Yes	Not stated	Yes	Yes	No
Hickman, 2021	United Kingdom	Yes	Yes	Yes	Not stated	Yes	Yes
Inan-Eroglu, 2018	Turkey	Yes	Not stated	Not stated	Not stated	Yes	Yes
Jarratt, 2002	United States	Yes	Yes	Yes	Yes	Yes	No
Karanges, 2019	Australia	Yes	Yes	Not stated	Not stated	Yes	Yes
Karim, 2019	Malaysia	Yes	Not stated	Not stated	Not stated	Not stated	No
Kunneke, 2018	South Africa	Yes	Yes	Not stated	Yes	Yes	No
Lake, 2019	South Africa	Yes	Yes	Not stated	Not stated	Yes	No
Levine, 1998	United States	Yes	Yes	Yes	Yes	Not stated	Yes
Ludwig, 2008	United States	Yes	Yes	Not stated	Not stated	Yes	No
Malhotra, 2019	Global [United Kingdom, New Zealand, United States]	Yes	Yes	Not stated	Not stated	Yes	No

**Author, year**	**Origin /Country of origin (where the source was conducted /published)**	**Experiences**	**Risks**	**Acceptability**	**Advantages**	**Strategies**	**Empirical data**
Margetts, 2009	England	Not stated	Yes	Yes	Not stated	Yes	No
Marks, 2011	United States	Yes	Yes	Not stated	Not stated	Yes	No
Martins, 2017	Global	Yes	Not stated	Not stated	Yes	Not stated	No
McInnes , 2007	Scotland	Yes	Yes	Yes	Not stated	Yes	Yes
Mialon, 2017a	Australia	Yes	Yes	Not stated	Not stated	Not stated	Yes
Mialon, 2016	Australia	Yes	Yes	Not stated	Not stated	Yes	Yes
Mialon, 2020d	Chile	Yes	Not stated	Not stated	Yes	Yes	Yes
Mialon, 2020a	Colombia	Yes	Not stated	Not stated	Not stated	Not stated	Yes
Mialon, 2020c	Colombia	Yes	Not stated	Not stated	Not stated	Not stated	Yes
Mialon , 2018	France	Yes	Not stated	Not stated	Not stated	Yes	Yes
Mialon, 2017b	France	Yes	Not stated	Not stated	Not stated	Not stated	Yes
Mialon, 2021	Latin America and the Caribbean	Yes	Yes	Yes	Not stated	Yes	Yes
Mialon, 2020e	South Africa	Yes	Yes	Not stated	Not stated	Yes	Yes
Morssink, 2001	United States	Yes	Not stated	Not stated	Not stated	Not stated	Yes
Murray, 2010	United States	Not stated	Yes	Not stated	Not stated	Not stated	No

**Author, year**	**Origin /Country of origin (where the source was conducted /published)**	**Experiences**	**Risks**	**Acceptability**	**Advantages**	**Strategies**	**Empirical data**
Nestle, 2015	United States	Yes	Yes	Yes	Not stated	Yes	No
Nestle, 2001	United States	Yes	Yes	Yes	Yes	Yes	No
Nestle, 1998	United States	Yes	Yes	Yes	Yes	Yes	No
Nestle, 2000	United States	Yes	Yes	Yes	Yes	Not stated	No
Nestle, 2013	United States	Yes	Yes	Yes	Yes	Yes	No
Olstad, 2013	Canada	Yes	Not stated	Not stated	Yes	Not stated	No
Oshaug, 2009	Norway	Yes	Yes	Yes	Yes	Yes	No
Palmer, 2009	United Kingdom [International]	Yes	Yes	Yes	Not stated	Not stated	No
Palmer, 2015	United Kingdom	Yes	Yes	Yes	Yes	Yes	No
PARN, 2017	United Kingdom	Yes	Yes	Yes	Yes	Yes	Yes
Peregrin, 2020	United States	Yes	Yes	Yes	Yes	Yes	No
Pereira, 2016	Brazil	Yes	Yes	Not stated	Not stated	Yes	No
Piaggio, 2020	Argentina	Yes	Yes	Yes	Not stated	Yes	Yes
Portman, 2016	United States	Yes	Yes	Yes	Yes	Yes	Yes
PotvinKent, 2020	Canada	Yes	Yes	Not stated	Not stated	Yes	Yes

Author, year	Origin /Country of origin (where the source was conducted /published)	Experiences	Risks	Acceptability	Advantages	Strategies	Empirical data
Reitshamer, 2012	United States	Yes	Yes	Yes	Yes	Yes	Yes
Rey-López, 2019	Spain	Yes	Yes	Not stated	Not stated	Yes	Yes
Richards, 2015	Australia	Yes	Yes	Not stated	Not stated	Not stated	Yes
Saboia, 2018	Portugal and Brazil	Yes	Not stated	Not stated	Yes	Yes	Yes
Serodio, 2020	United States	Yes	Not stated	Not stated	Not stated	Not stated	Yes
Shafer, 1996	United States	Yes	Not stated	Yes	Yes	Not stated	No
Scheffer, 2015	France	Yes	Yes	Yes	Yes	Yes	Yes
Simon, 2013	United States	Yes	Yes	Yes	Yes	Yes	No
Simon, 2015a	Australia	Yes	Yes	Yes	Yes	Yes	No
Simon, 2015b	United States	Yes	Yes	Yes	Not stated	Yes	No
Smith, 2014	United Kingdom	Yes	Yes	Yes	Yes	Yes	Yes
Steele, 2020	United States	Yes	Yes	Not stated	Not stated	Not stated	Yes
Stein, 2017	United States	Yes	Not stated	Not stated	Yes	Not stated	No
Stein, 2015	United States	Yes	Yes	Yes	Yes	Yes	No
Stover, 2017	United States	Not stated	Yes	Not stated	Not stated	Yes	No

Author, year	Origin /Country of origin (where the source was conducted /published)	Experiences	Risks	Acceptability	Advantages	Strategies	Empirical data
Tanrikulu, 2020	United States	Yes	Yes	Not stated	Not stated	Yes	Yes
Tappenden, 2015	United States	Yes	Yes	Yes	Not stated	Yes	No
Tobin, 1992	United States	Yes	Yes	Yes	Yes	Yes	No
Unknown, 2005	United States	Yes	Yes	Yes	Yes	Yes	No
Unknown, 2000	United States	Yes	Yes	Yes	Yes	Yes	No
VanTulleken, 2018	United Kingdom [International]	Yes	Yes	Yes	Not stated	Yes	No
Wallace, 2014	United States	Yes	Yes	Not stated	Not stated	Not stated	Yes
Wilkins, 2020	United States	Yes	Yes	Yes	Yes	Yes	No
Wilkins, 2010	United States	Yes	Yes	Not stated	Not stated	Not stated	No
Wilkins, 2009	United States	Yes	Yes	Yes	Not stated	Not stated	No
Woteki, 2006	United States	Yes	Yes	Yes	Not stated	Yes	No
Wynne , 2003	New Zeland	Yes	Not stated	Yes	Yes	Yes	No
