# Supplementary material for: Interactions Between Nutrition Professionals and Industry: A Scoping Review
Source: Int J Health Policy Manag. 2023 Aug 22;12:7626. doi: 10.34172/ijhpm.2023.7626 (PMC10590255; doi:10.34172/ijhpm.2023.7626)
Supplement: Supplementary file 3 — Codebook With Illustrative Quotes – Nutrition Professionals Experiences of Industry Interactions and Acceptability. [file ijhpm-12-7626-s003.pdf]

Article title: Interactions Between Nutrition Professionals and Industry: A Scoping Review

Journal name: International Journal of Health Policy and Management (IJHPM)

Authors' information: Virginie Hamel[1,2]*, Marita Hennessy[3], Mélissa Mialon[4], Jean-Claude Moubarac[1,2]

[1]Department of Nutrition, Faculty of Medicine, University of Montreal, Montreal, QC, Canada.

[2]Centre de recherche en Santé publique, Montreal, QC, Canada.

[3]College of Medicine and Health, University College Cork, Cork, Ireland.

[4]Trinity Business School, Trinity College Dublin, Dublin, Ireland.

***Correspondence to**: Virginie Hamel, Email: virginie.hamel.1@umontreal.ca

Citation: Hamel V, Hennessy M, Mialon M, Moubarac JC. Interactions between nutrition professionals and industry: a scoping review. Int J Health Policy Manag. 2023;12:7626. doi:10.34172/ijhpm.2023.7626

Supplementary file 3. Codebook With Illustrative Quotes – Nutrition Professionals Experiences of Industry Interactions and Acceptability

Code	Description	No. of documents	Select illustrative quotes
**Nutrition Professionals (NP) experiences of industry interactions**			
**Interactions with individual NP**	NP interacting directly with industry		
**Active (solicited or implying involvement from individual)**	Includes interactions in which NP is actively involved in/engaged		
Employment by or consultation service for industry	NP have a part-time job/role in an industry setting while having another job, the nature of which could be consultation/advisory board roles	8	There are many employment opportunities for dietetic professionals and it's quite easy to have multiple part-time activities," explains Lacey. "You could be working for a food company and at the same time be working part-time for a health care organization. The challenge is to separate those two roles so that you don't let the interest of one of your employers inappropriately influence the responsibilities of your other job. (Peregrin, 2020)<br><br>The relationships span a wide range of common practices in which companies provide free samples, […], provide consulting contracts, […]. (Woteki, 2006)
Having stock holding or ownership in an industry	NP have stocks in food company or own their own food business	2	In other cases, dietetics practitioners may have greater financial stakes in companies through stock holdings or ownership. (Woteki, 2006)
**Endorsement of company and its brands**	Interactions that might represent an endorsement of industry products or the industry itself.		

Code	Description	No. of documents	Select illustrative quotes
Endorsement of products or industry in the public sphere	NP engages in marketing in the media (social media, magazine, or traditional media) in partnership with industry– with or without financial compensation	6	Both RD and non-RD bloggers had sponsored posts, providing bloggers with free products and/or financial compensation in exchange for reviewing the products. Bloggers also shared affiliate links for which they received commission for purchases made through the link. Both food and non-food items were promoted, for example, cookware, clothing, books, and meal delivery services. (Chan, 2020)
Endorsement in private practice or in the office	NP directly sell products like supplements	4	RD is offered compensation from a company if she recommends their product to her clients (ex. home delivered prepared meals) (51.6% in FU survey). (Fornari, 2001)
Endorsement and/or co-creation of educational material	NP develop educational material with industry and/or endorse/use/promote industry material	3	Seventy-four percent were Registered Dietitians. Only 9% were currently enrolled in a nutrition program. The largest majority of respondents knew about or had worked with the National Dairy Council's (NDC) materials (90%) and Campbell's label collection program (84%). A smaller majority knew about or had worked with the Beef Industry Council's guide (68%), Dole's supermarket tours (65%), and Pizza Hut's reading program (53%). […]. (Levine, 1998)<br><br>Most dietitians are involved at one time or another in the planning of educational materials for their patients, students, or the general public. Within this group, there are dietitians that work directly for health care organizations while others work for food, beverage, supply, or equipment manufacturers and suppliers that benefit financially when their products are promoted. (Unknown, 2005)
**Other direct interactions**	Industry directly interacts with NP that haven't actively sought or partnered in		
Receiving travel and conference attendance funding	NP receive funding from industry to attend meetings/ conferences; can include fees, travel, etc. (source not specified, whether it was via university, work setting or professional body)	10	Since then, I have discussed product development at meals hosted by P&G officials, explained my views of olestra to company staff ($500 honorarium), and co-chaired a session and was lead author of a report[3] from a P&G-sponsored conference on fat-modified foods28 (travel and meals, $4000 honorarium). (Nestle, 1998)<br><br>One participant in our interviews explained that the industry also often pays for the travel and fees for students and academics to attend these conferences [academic]. (Mialon, 2020a)
Continuing education directly provided by industry	Industry sponsors/provides continuing professional education to NP	5	Actors in the food industry also built alliances with health professionals in nutrition and dietetics. Nestlé and the SASA sponsored the ''Continuing Nutrition Education (CNE) Programme for Dietitians and Nutritionists'' in 2019 [A66 (Mialon, 2020e)<br>Proliferations of CPE "freebies" mentioned frequently by these RDs, which are often produced by commercial companies. These freebie courses are usually 1-hour online webinars, usually produced by commercial companies, often food or beverage related. Traditional CPE providers, which have been universities or other agencies, have needed to charge to support the cost of the CPE, usually produce longer, more intensive, or more in-depth educational experiences. This shift in type and sponsorship has implications for the dietetic profession if more CPE is provided by for-profit companies. (Wallace, 2014)
Receiving targeted communication from industry (e.g., via letter/email)	NP receiving targeted communication from industry by email or letter	3	Using reputable bodies to advance the science supportive of industry products: In early 1998, the Australian Sugar Industry's (ASI) Nutrition Consultant wrote to key food and nutrition professionals and educators to announce that the ASI would launch a new campaign promoting sugar in moderation (Australian Sugar Industry, undated). (Dixon, 2004)

Code	Description	No. of documents	Select illustrative quotes
			More than half of the respondents learned about the programs through direct contact by industry (62%), resource catalogues (57%), professional colleagues (56%), or by word-of-mouth (55%). (Levine, 1998)
**Interactions with professional body or nutrition and health organizations**			
**Nutrition professional education**			
**Industry involvement in NP events/conferences**	Interactions around NP education		
Industry participation in scientific event(s)	Industry participates in scientific events/hosts symposia via sponsored/affiliated speakers, holding conference sessions/symposia, etc.	22	Of the 34 scientific sessions offered by ASN, each consisting of three to four panelists, 6 were supported by PepsiCo, and 3 were supported by the Mead Johnson Pediatric Nutrition Institute (Mead Johnson is one of the largest manufacturers of infant formula). Other supporters, with one session each, included the Egg Nutrition Center, Kellogg, DuPont Nutrition and Health, Ajinomoto, and the National Dairy Council. Examples of specific sessions sponsored by PepsiCo are below. (Simon, 2015)<br><br>Four of these companies belonging to the food and beverage branch were responsible for the four satellite symposia held at the event, establishing a structural and direct relationship between sponsorship and inclusion in the scientific program. The satellite symposia took place at lunchtime or in the evening and included boxed lunches/dinners and "happy hours"5 [5 Free alcoholic and non-alcoholic drinks and hors d'oeuvres.] along with presentations on topics of interest to the sponsoring companies, namely: 1)"Strategy for the adoption of healthy lifestyles"; 2) "Understanding the action of calcium and vitamin D on bone health"; 3) "Major food sources of sodium—targets for a strategy to reduce consumption"; and 4) "Soybeans—from allergy and intolerance to a healthy option: an update on childhood diets. (Canella, 2015)<br><br>Kathi Eckler, RD, Kellogg Company's director of nutrition marketing, Frozen Food and Snacks, presents at the Student Breakfast at the 2012 Food & Nutrition Conference & Expo in Philadelphia, PA. Kellogg Company is an Academy sponsor. (Stein, 2015)<br><br>Also, FNCE is attended by many students and future RDs, thus exposing upcoming generations of professionals to this corporate influence. (Simon, 2013)
Exhibit booths	Industry holding exhibits during nutrition-related scientific events – either in the conference rooms, or other room, separated from these. Here, industry can meet NP and future NP and gives samples, brochures, or research updates	17	As ADA makes you more visible, you have the right and the responsibility to do the same. Interact and network with exhibitors at events such as the Food & Nutrition Conference & Expo. Learn about upcoming programs and initiatives and the part you might play in them. Ask, "What's in it for me?" You may be pleasantly surprised at the professional and personal opportunities that present themselves. (Diekman, 2007)<br><br>It's a truly surreal experience just to walk into the expo hall. You know it's supposed to be a nutrition conference and yet it feels like a food industry event. Junk food expo is really the best descriptor. As you walk in, all you can see are the massive booths of companies like Coca-Cola, Hershey's, and PepsiCo. Once you get past those, you have to walk toward the back to see any "real food" booths, which are much smaller, such as the California Walnut Board. Then further back, tucked away in a special pavilion, are the

Code	Description	No. of documents	Select illustrative quotes
			organic and “natural” companies, such as Amy’s Kitchen, Mary’s Gone Crackers, and Manitoba Harvest (hemp seeds). (Simon, 2013)
Industry promotional and educational materials distribution	Industry promotional/educational materials (e.g., education packet, flyers and brochures) are distributed to/available for NP during scientific events	8	THIS CONFERENCE TOTE BAG & BOOKLET BROUGHT TO YOU BY… Every year upon arriving at FNCE, attendees pick up a complimentary tote bag that contains general conference information booklet as well as 'educational materials' provided by the Academy's partners (to clarify: Abbott Nutrition, The Coca-Cola Company, and the National Dairy Council are Academy partners; General Mills, Kellogg's, PepsiCo, Soyjoy, and Unilever are Academy premier sponsors; Alaska Seafood, Campbell’s, ConAgra Foods, Del Monte, Haas Avocado Board, Jamba Juice, Nature Made, and Safeway were this year’s FNCE sponsors). (Dietitians for Professional Integrity, 2013)<br><br>After completing the required Congress registration, all of the participants crossed a hall arranged for commercial exhibition of the sponsoring companies in order to arrive at a desk (as shown in Figure 1) where the staff handed out a bag containing the map of the premises, a summarized programme of the whole event, a notepad, a ballpoint pen, and two flyers of Unilever, a company that did not have any commercial stand. On the provided bags, the name of one of the sponsors that had a stand was printed, a company producing dietary supplements (Herbalife). (Piaggio, 2020)
Provision of meals /beverages/ food samples	Industry offers meals, beverage, or food samples to participants during nutrition events	8	Furthermore, the companies producing foods offered free samples. The result was that during the intervals of the scientific programme, the commercial area was packed with people going over the stands and consuming free samples (yoghurts, flavoured milks, bottled water, coffee, cereals, cereal bars, crackers, etc.) (Figure 4). (Piaggio, 2020)<br><br>Even though this activity had already been included in the official program, a special invitation was distributed to event attendees, with information on special attractions such as the distribution of boxed lunches/dinners and raffle giveaways. (Canella, 2015)
Sponsorship of nutrition event /conference	Financial support for a whole scientific conferences/event	22	The 21st IUNS Congress was held in Buenos Aires, Argentina, in 2017, in a five-star hotel located in the financial district. There were 3,038 participants. The scientific programme comprised 24 satellite symposia, 6 plenary lectures, 40 special lectures, 108 parallel symposia, and 28 sponsored symposia. It was sponsored by large companies producing foods, additives, diet supplements (International Union of Nutritional Sciences, n.d.). (Piaggio, 2020)<br><br>The field of nutrition provides some good examples of the influence of the food and beverage industries in health scientific events. The case presented here was a biannual national congress held in Brazil. In 2011, the Brazilian Society of Food and Nutrition (Sociedade Brasileira de Alimentação e Nutrição, SBAN) (São Paulo), a scientific entity in the field of nutrition, organized a congress on “evidence-based nutrition” that aimed to “expose how scientific evidence should be determined.” Reasoning that it would not be feasible to hold an event without sponsorships, support from companies was sought during its organization and then categorized according to the invested amounts. A world-renowned corporation and manufacturer, retailer, and marketer of nonalcoholic beverage concentrates and syrups was ranked as a “diamond” sponsor, the highest category of financial contribution, while three other transnational food and beverage companies and two pharmaceutical companies were classified as “gold” sponsors. (Canella, 2015)

Code	Description	No. of documents	Select illustrative quotes
**Continuing education provided/ sponsored by industry**	NP can access continuing professional education sponsored or developed by industry through their professional body or health and nutrition organizations	6	The following continuing education session is available for purchase by RDs directly from AND's website: Restaurant Menu Labeling: Impact on the Environment of Nutrition and Dietetics.16 The two presenters are from the National Restaurant Association, the powerful lobbying organization for the restaurant industry, which fought for decades against menu labeling; thus having them present on this topic seems especially disingenuous. (Simon, 2013)<br><br>The NZDA recently worked with Heinz Watties in New Zealand to organise a series of seminars aimed at dietitians and other health professionals, which pro- moted the consumption of fruits and vegetables. Follow- ing the seminars the NZDA worked with Heinz Watties to produce a series of fact sheets with nutritional infor- mation on fruits and vegetables and ideas for increasing consumption. (Wynnes, 2003)
**Partnership and sponsorship**			
Partnership on programs or advocacy	It includes co-construction, endorsement or revision of educational material(s) created with or by industry (e.g., fact sheets) or partnership with industry to build and develop nutrition programs. Could be sponsored partnership. Includes collaboration on activities.	39	The education-oriented CSR initiatives included funding for EatTracker (n = 1), a consumer resource developed by Dietitians of Canada that helps track nutrient intake (EATracker": "Nestlé Canada wants to help Canadians make the right food and activity choices. EATracker is a tool produced by the Dietitians of Canada (and developed with a technical grant from Nestlé Canada) to help individuals keep track of their daily food and activity choices and show how they are doing compared to current recommendations."). (Potvin-Kent, 2020)<br><br>Partner status gives the right to co-create and co-brand an Academy-themed 'information consumer campaign', like the Coca-Cola 'Heart Truth Campaign' involving fashion shows of women wearing dresses coloured the Coke red, as modelled above by Sylvia Escott-Stump. (Gomes, 2013)
Sponsorship of professional bodies and health and nutrition organizations	Sponsorship of association and professional body for their general activities, including long-term sponsorship and corporate membership, but excluding conference sponsorship and sponsored partnerships for specific activities	29	In March 2008, the ADA announced that the Coca-Cola Company had become an "ADA Partner" though its corporate relations sponsorship program. In promoting this program, the ADA states that becoming an ADA partner "provides partners a national platform via ADA events and programs with prominent access to key influencers, thought leaders and decision makers in the nutrition marketplace" (American Dietetic Association 2008). (Brownell, 2009)<br>Of the nine associations in which the existence of an ethical code or declaration of interests could be established, five (56%) enjoyed some type of sponsorship: in the case of the remaining 16, 10 (62.5%) enjoyed some type of sponsorship (odds ratio: 0.75; 95% confidence interval: 0.14-3.94). A total of 64% of the societies and foundations studied reported receiving some type of sponsorship from food companies that market unhealthy products (Table 2). This percentage was highest for pediatric societies, with a figure of 83%, and nutrition societies, with a figure of 80%, whereas none of the epidemiology and public health societies reported receiving any sponsorships from food and drink companies. The leading food industry companies which sponsored scientific societies and foundations active in the field of childhood nutrition and obesity in Spain were Nestlé, which sponsored 36%, Coca-Cola which sponsored 32%, and Danone which sponsored 24% of the societies and foundations studied (Table 3). […]. (Guzman-Caro, 2020)

Code	Description	No. of documents	Select illustrative quotes
**Other interactions**	Professional bodies provide industry access to NP through their actions (e.g., endorsement), their communications (e.g., emails), their activities (e.g., networking opportunities) and their structure (e.g. committee members with ties)		
Advertising through journal(s), direct mailing, and website	Industry may have a page of advertising in a dietetic/nutrition professional body's scientific journal; professional body may give details (including email addresses) of their members to industry to enable them to send publicity directly to them (or they may do so on industry's behalf); industry advertising may be seen on the website(s) of professional bodies	17	Acceptance by professional bodies of corporate sponsorship for events and journals is commonplace. The inside covers of journals such as the Australian Journal of Nutrition and Dietetics and the Asia Pacific Journal of Clinical Nutrition attest to this. It is hard to say what subtle influences these arrangements may or may not give rise to. Neverthe-less, there does appear to be a growing trend for nutritionists and others to serve as 'third party' defenders of food products or industry interests. (Dixon, 2004)<br><br>Industry sources pay $20,000 per fact sheet to the ADA and take part in writing the doc-uments; the ADA then promotes them through its journal and on its website. (Brownell, 2009)
Endorsement of food products (such as food certi-fication)	Endorsement of food products by professional body/organization (e.g., food certification). It includes explicit or implied endorsement	5	Under the headline Diet Message Funded by Food Giants (McKenzie, 2001), some background was provided to a community service announcement from the Dietitians As-sociation of Australia, in which the association's president argued that breakfast cereal could improve student exam results. There was no mention that the Australian Dairy Corporation paid for the advertisement's production. Nor was there any mention that ce-real manufacturers are 'generous' supporters of the association, which has over 2000 members. (Dixon, 2004)<br><br>Another type of relationship with food companies is one in which the nutrition profes-sional group certifies that the nutrient composition of a product meets the society's nu-tritional standards, and these results are then often publicized by the company. An ex-ample of such a collaboration is that of the American Diabetes Association with various food companies. If, after analysis, the food product is found to meet their standards, the company is per- mitted to advertise on the product that it is "A Proud Sponsor of the American Diabetes Associa- tion," with the association's accompanying logo. Approxi-mately 20 foods from at least four compa- nies currently appear in the market with such a statement. (Tobin, 1992)
Prizes and awards sponsored by or from industry	Industry confers prizes and awards to NP, including those at conferences/events	5	Prizes and scholarships from the food industry were also awarded at the XVIII Colom-bian Congress of Nutrition and Dietetics (one from Boydorr and one from ILSI) and the Congress of the Latin American Society of Nutrition (SLAN) held in Mexico in 2018 (from Vitanui and Yakult). (Mialon, 2020b)<br><br>Dairy Campaign Wins Dietitian DAA Award At its annual meeting in May 2104, the DAA issued its President's Award to Kim Tikellis, dietitian and nutrition manager at dairy giant Fonterra. According to DAA, she "won the award for the innovative cam-paign which has empowered 2,000 Fonterra employees around Australia to deliver healthy food messages to everybody, anywhere, every day, especially in relation to the core dairy food group."30 (Simon, 2015a)
Leaders or committee members with industry ties or affiliation	Members and leaders of professional bodies may have ties with industry actors	3	In June 2005, the House of Delegates (HOD) of the American Dietetic Association (ADA) surveyed affiliate and delegate leaders to obtain information about how these or-ganizational units utilized and viewed industry relations.2 Of the 82 leaders who were asked to complete the brief survey form, 39 (48%) responded, and this reflect- ed data from 22 affiliate leaders and 17 leaders of dietetic practice groups (Unknown, 2005)

Code	Description	No. of documents	Select illustrative quotes
Networking opportunities	Industry creates or supports networking opportunities within the profession through professional bodies	3	Financial arrangements among entities with clear financial interests in activities convened and/or endorsed by professional scientific groups (e.g., support of ASN's annual meeting and satellite gatherings, prizes, membership networking events, fellowships, travel awards, and other similar activities). (Garza, 2019)
Industry-led surveys and focus groups with members	Industry access to NPs through professional body by doing survey or focus group with them	2	A Hershey's partnership with the American Dietetic Association offers an online survey of registered dietitians that promotes the daily inclusion of chocolate as part of a "balanced lifestyle. (Freedhoff, 2011)<br><br>Abbott Nutrition has obtained feedback and advice from the BDA membership via focus groups and surveys. Danone has consulted with the BDA membership in relation to some of their produces via regional groups, focus groups, and surveys. Both corporate members advise that through their partnership with the BDA they are able to have frank and open conversations with groups of healthcare professionals, and work together to feed back into the business (British Dietetic Association, 2017)
**Interactions with or within educational institution (e.g., University)**	Interaction with or within university or college where future NP received their training		
Provision/sponsorship of student educational materials/activities/events/internships	Interactions/activity inside the classroom, activity organized by school that imply industry (visit food expo with the class), branded Materials used in the classes, conferences held by industry for students	6	At the University, the advertising occurs mainly through sponsorship of events and research funding, donation of materials and structures and publicity in the classroom. (Pereira, 2016)<br><br>In 2019, ILSI Nor-Andino organised two workshops on FOPL: one in March, targeted at BSc students in nutrition and dietetics from the Faculty of Sciences of the Pontificia Universidad Javeriana [A127]. (Mialon, 2020c)
Scholarship, sponsorship awards and prizes for students	Industry provides scholarship, funding, awards, and prizes for students in an educational context	4	In addition, since 1998, Nestlé sponsored a scholarship for the students of INTA, which current objective is to "contribute to the academic and institutional development of INTA, through the financial support of maintenance for young researchers who show interest and potential to start an academic career" [A87 Nestlé]. (Mialon, 2020d)
Sponsored continuing education	The educational institution provides professional continuing education sponsored/held by industry for NP	2	First Conference on Sweeteners was held in Santiago, a talk for professionals convened by the Nutrition and Dietetics Program at the Universidad Santo Tomás." [A52 Coca-Cola] "[In the] School of Nutritionists […] they defend sweeteners, they have classes about sweeteners everywhere, they travel around the country talking about sweeteners." [member of government]. (Mialon, 2020d)
**Other channels**	NP can interact with industry through society, job setting, foundations, or industry website		
**Interactions marketing/interactions in work setting**	Interactions that could occur in settings where NP work (e.g., hospitals, universities, community)		
Sales representative visits	NP are being approach by sales representative at their workplace	3	A nutritionist in private practice is consistently approached by supplement companies to sell their products in the office, the nutritionist will reap financial gains when their supplements are sold (29.0% in FU survey). (Fornari, 2001)<br>22/50 dietitians have been visited by BMS personnel - The main purpose of these visits was to provide product information, educational updates, or infant nutrition support. (Mc Innes, 2007)

Code	Description	No. of documents	Select illustrative quotes
Gifts, samples, and educational material	NP receiving gifts like toys, calendars, posters, diary convers, stationery, money-off vouchers, bags, pens, etc.	2	38/50 have received incentives from BMS personnel. - receiving advertising through gifts (stationery, diary covers, calendars/posters, developmental toys, meals, coffee and money-off vouchers). (Mc Innes, 2007)
Attending lunchtime meeting (industry representative speaking)	NP receiving support to have company attend lunchtime meetings (e.g., to update product knowledge)	1	Members are frequently offered the following sponsorship support from companies: Funding your attendance at professional or scientific events; Attending your department's lunchtime meetings to update product knowledge. (British Dietetic Association, 2017)
Industry marketing	NP are exposed to industry marketing in their work setting	1	And another area of ethical concern, especially to these RDs who promote breastfeeding, was the prevalence of marketing of infant formulas. Riley explained: Well, pharmaceutical companies' market at the hospital. And maybe sometimes the type of formula they give, that's the type formula they [the family] feel is best, and you have to really explain to them about the composition of the formula, that it is a good choice, but, you know, with breastfeeding being the highest choice as far as nutritionally, for the infant. And our [WIC] program has a contract, and it may be [for] a different formula. And you're not saying the formula [from the hospital] is not good, because nutritionally the nutrients are in there, but you just have to explain it to them. (Wallace, 2014)
Interactions through colleagues or other professionals	Colleagues and/or other professionals (e.g., physicians) could be channels through which NP interact with or experience influence from industry actors (e.g., NP received products information from a NP working in industry or NP asked by a physician to promote weight loss program using pills and supplements)	3	RD asked by a physician to promote a weight loss program using pills and supplements, which the RD does not support (61.3% in FU survey); RD in private practice reports another professional as trying to convince her to market own line of supplements in office instead of referring to health food store (17.7% in FU survey). (Fornari, 2010)<br><br>The College frequently receives inquiries about potential conflicts arising from RDs who work in the food industry. Registered Dietitians in this sector provide product information to the public and nutrition professionals such as other RDs. Where RDs are endorsing or recommending products aimed towards the public, a conflict of interest may exist. (Cohen, 2009)
Industry website, media advertising and promotional events	Educational materials and information for professional and consumers created by industry and delivered via the web/television advertising or other promotional events (e.g., industry had a specific website for health professionals and publishing educational materials for professionals and fashion show organized by a food industry)	5	Nestlé has a specific website for health professionals (A354). ANIA and Nestlé also published educational materials for professionals, parents and schools. (Mialon, 2018)<br><br>SASA, on its websites, also proposed education material for children between 9 and 13 years old and for health professionals. (Mialon, 2020e)
Foreign aid	NP can interact with industry through foreign aid, including the provision of products to alleviate malnutrition	2	Critics of Big Food involvement with nutrition congresses make an even more basic mistake, when they assume that the nutrition profession in general is aware of and opposed to the ill-effects of transnational Big Food corporations on public health. This is not the case. Influential nutritionists concerned with the alleviation of undernutrition in the South, work with corporations whose products provide 'quick energy' to undernourished children. (Gomes, 2013)
**Acceptability**			
Acceptable	Interactions with industry are acceptable. Includes citation mentioning that it is inevitable or normalized to have such	21	"One member said, "I think a lot of things can't get done alone…you need partners." She went on to say that "it takes people, it takes resources, it takes time, and it takes

Code	Description	No. of documents	Select illustrative quotes
	interactions, which implied acceptability		brains to pool resources together to get things done," implying that corporate sponsors were a necessary part of this equation.". (Smith, 2015)<br><br>"Not all dietitians believe the corporate sponsorship issue is problematic. "I believe this is a tempest in a teapot," says Neva Cochran, MS, RDN, LD, a nutrition communications consultant who's held many positions within the Academy, including past president of the Texas Dietetic Association and member of the board of directors. "While there's perceived controversy because a few very vocal people, including nonmembers and non-RDNs, are working hard to create it, I don't believe that there's actually that much real controversy," Cochran says." (Palmer, 2015)
Encouraged	Encouraged and presented as positive, helpful, and useful. Industry is viewed as an ally in promoting public health	25	"As Sophie explained: They're [industry] a lot louder, they're a lot more involved. In some ways people are listening to them a lot more than they're listening to us…So we need to partner with them so we are sure that we are capturing the largest audience we can. And so we need to be more engaged with them. RDNs in this camp viewed their ability to enact change by working within the existing system. […] Because of this, it is important for public health practitioners to engage with and provide expert advice to industry (Wansink & Peters, 2007).". (Portman, 2016)<br><br>The ADA encourages the government, food manufacturers, food commodity groups, and qualified food and nutrition professionals to work together to inform consumers about this new technology and encourage availability of these products in the marketplace. (Bruhn, 2016)
**Conditional acceptability**	Acceptable under certain conditions		
Depend on industry type	Interactions are acceptable with some types of industry, but not for others	7	"I agree with DFPI that the problem at hand is not collaboration with the private sector, but rather who in the private sector we align with. […] I strongly believe that companies aligned with the Academy should support practices for reducing the prevalence of obesity and disordered eating and protecting global environmental welfare. […]." David Wiss, MS, RDN, CPT. (Dietitian for Professional Integrity, 2013)<br><br>"Reitshamer, Schrier, Herbold, and Metallinos-Katsaras (2012) surveyed Academy members' opinions about corporate sponsorships to understand the organizational practice in more detail. The authors asked Academy members to rate the Academy's current corporate sponsors based on their perceived "acceptance" level. They found that a majority of members felt three of the thirteen companies were "unacceptable" as Academy sponsors: Coca-Cola, PepsiCo, and Mars, Inc. The other sponsors, including Unilever, Kellogg Company, and General Mills, were perceived as "acceptable" sponsors by the majority of respondents.". (Smith, 2014)
Depend on the nature of the interaction type	Some interactions are acceptable, while others are not, depending on the type of interaction (e.g., funding specific sessions or speakers is not acceptable, but sponsoring congresses is)	5	"Some nutritionists commentating on the topic believe that industry contributions in the form of travel, meals, honoraria, and conference sessions pose minimal conflict, but that ethical issues become more acute when companies contribute toward specific academic and professional programmes[65]. Others argue that the public assumes that non-profit professional associations provide unbiased, objective information about diet and health, and that sponsorship implies product endorsement.". (Nestle, 2001)

Code	Description	No. of documents	Select illustrative quotes
Other conditions	Some interactions are acceptable, but at some condition (e.g., acceptable if the code of ethics is followed)	11	“Collaboration between members of the Academy of Nutrition and Dietetics (Academy) and the food and nutrition industry, as well as members of academia, can result in innovative research that fills the gaps in the science of food and nutrition. These relationships—when established under the standards outlined in the Code of Ethics for the Nutrition and Dietetics Profession and in the Academy’s six scientific integrity principles—can safeguard against potential conflicts of interest (COIs) and ensure that all decisions are made in the best interest of the patient or client.”. (Peregrin, 2020)
Not acceptable	No interaction is acceptable	22	“Stuart Gillespie was outraged by the penetration of Big Food in congresses held in association with the International Union of Nutritional Sciences. ‘The IUNS needs to explain why it continues to accept sponsorship from companies whose practices and products have consistently been shown to damage human health and nutrition. Why spend so much time and effort organising yet more scientific sessions on the drivers of the obesity epidemic when some of the main causes are sitting in the audience?’. (Gomes, 2013)<br><br>“The Academy of Nutrition and Dietetics' recent history of food industry ties has resulted in some members publishing ‘statements of concern’,. These dietitian-penned accounts shed light on why some dietitians renounced their memberships. Many of these first-person pieces call for sponsorship reform and question whether the Academy’s actions align with its mission (‘a world where all people thrive through the transformative power of food and nutrition’) and vision (‘accelerate improvements in global health and well-being through food and nutrition’). Dietitian for Professional Integrity is a dietitian-organized grass-roots professional community that opposes The Academy’s longstanding and consistent alignment with purveyors of highly processed, minimally nutritious foods and beverages. Created in February of 2013, the group has advocated for sponsorship reform within the Academy.”. (Bellatti, 2019)
