# Supplementary material for: Interactions Between Nutrition Professionals and Industry: A Scoping Review
Source: Int J Health Policy Manag. 2023 Aug 22;12:7626. doi: 10.34172/ijhpm.2023.7626 (PMC10590255; doi:10.34172/ijhpm.2023.7626)
Supplement: Supplementary file 4 — Codebook With Illustrative Quotes – Perceived Advantages and Risks. [file ijhpm-12-7626-s004.pdf]

Article title: Interactions Between Nutrition Professionals and Industry: A Scoping Review

Journal name: International Journal of Health Policy and Management (IJHPM)

Authors' information: Virginie Hamel[1,2]*, Marita Hennessy[3], Mélissa Mialon[4], Jean-Claude Moubarac[1,2]

[1]Department of Nutrition, Faculty of Medicine, University of Montreal, Montreal, QC, Canada.

[2]Centre de recherche en Santé publique, Montreal, QC, Canada.

[3]College of Medicine and Health, University College Cork, Cork, Ireland.

[4]Trinity Business School, Trinity College Dublin, Dublin, Ireland.

***Correspondence to**: Virginie Hamel, Email: virginie.hamel.1@umontreal.ca

Citation: Hamel V, Hennessy M, Mialon M, Moubarac JC. Interactions between nutrition professionals and industry: a scoping review. Int J Health Policy Manag. 2023;12:7626. doi:10.34172/ijhpm.2023.7626

Supplementary file 4. Codebook With Illustrative Quotes – Perceived Advantages and Risks

Code	Description	No. of documents	Select illustrative quotes
**Perceived advantages**			
**Advantages for organizations**			
Financial survival	Interactions and partnerships with industry are seen as a way to survive financially	15	And yet, Polly observed the frustrating reality that is the need for funding. While some nonpartisan, public-sector funding does exist for research, it would be nearly impossible to advance science fully without private-sector support and partnerships, she said. (Boyce, 2017)<br><br>Others brought up issues of scale and suggested that an organization as large as the Academy—with over 75,000 members— needs corporate sponsors to "sustain" itself. (Smith, 2014)
Better fulfil mission	Industry partnerships/support can enable organizations to better fulfil their mission (e.g., by facilitating/funding educational programs that could not otherwise have happened)	6	Finally, in accepting the statement that all foods can be part of a healthful diet, we can be confident about working with partners, such as food and pharmaceutical industries, to further our nutrition education efforts through their funding of projects that would otherwise be unfunded by ADA. (Derelian, 1996)

Code	Description	No. of documents	Select illustrative quotes
			Engagement with industry should help to fulfil the purposes of nutrition research, public health nutrition programmes and activities, and public policy and programme development, in order to prevent nutrition-related damage to public health and to promote consumption of nutritionally adequate healthy diets. Such initiatives should accept and follow the food and nutrition priorities of national governments, independent professional bodies, and those of UN organisations such as those embodied in the WHO/UNICEF Global Strategy on Infant and Young Child Feeding[1] and the WHO Global Strategy on Diet, Physical Activity and Health[2]. (Oshaug, 2009)
Additional income (for dinner, for 5-star hotel lifestyles, etc.) and business growth/profile	Sponsorship and funding can allow additional income to hold events in prestigious location and/or to offer more activities within congress. Strategic alliances are also adopted as a primary means for accelerating business growth	4	She believes that industry support was essential to provide speakers' dinners and other social activities. 'I am sure the social activities for speakers, in general, could not have been mounted without the support of industry funds, and especially in this economic climate in Spain'. If the rumour that the surplus of the Granada ICN amounts to somewhere around $US 750,000 ismore or less accurate, her assumption might not be correct. (Gomes, 2013)
**Advantages for professionals and organizations**	Advantages that apply to both the professional and its organization		
Public outreach	Interactions help professional bodies/organizations, and its members reach the public with their message and education programs	8	The corporate sponsorship program, under its current structure, is described (per a 2012 Academy annual report) as a means to work with industry "to build awareness of the Academy and its members, to share science-based in-formation and new research with Academy members, and to enable [the Academy] to reach millions of con- sumers with healthy eating messages." (Stein, 2015)<br><br>They're [industry] a lot louder, they're a lot more involved. In some ways people are listening to them a lot more than they're listening to us...So we need to partner with them so we are sure that we are capturing the largest audience we can. And so we need to be more engaged with them. (Portman, 2016)
Benefit from industry's expertise	Industry's marketing and public relations expertise can benefit NP	5	Working together, food industry partners can provide resources and tools that dietitians can use to have a bigger impact.". (Palmer, 2015)<br><br>Reasons to work with food, drink and associated companies include receipt of their support and resources and influencing their policies and activities. Industry skills, experiences, resources and networks may indeed potentially be harnessed. (Oshaug, 2009)
Build awareness of the organization and its members	Interactions allow more public visibility for the professional and its members	4	Corporate sponsorship enables the Academy—as it does for nonprofit organizations and associations nationwide—to build awareness of the Academy and our members; to share science-based information and new research with members; and to enable the Academy to reach millions more

Code	Description	No. of documents	Select illustrative quotes
			consumers with omessages than would otherwise be possible. ("Truth and transparency," 2013, para. 4) (Smith, 2014)<br><br>Collaboration with industry is likely to continue as opportunities arise and is viewed as being beneficial in terms of promoting dietitians and the NZDA, and ensuring that information distributed is accurate, balanced and up to date. (Wynne, 2003)
Enhance credibility and reputation	Interactions and partnership with industry contributes to credibility, growth, and a better reputation	2	Organizations in the private sector offer […], credibility, and, often, strong networks for message dissemination, all of which provide significant added reach to our key messages. (Derelian, 1995)
**Advantages for individual professionals**			
Continuing education and information	Industry provides NP with education and information (e.g., information about products)	11	Feedback was obtained from students, observers, course facilitators and tutors, the Director and staff of the Department of Medicine at the University of Indonesia, and the GTZ team leader. The involvement of the food industry was significant, helping the students to appreciate that scientific knowledge alone was not enough and that it had to be combined with technical knowledge and skills. (Crowther, 1999)<br><br>These RDNs viewed the Expo as a conduit for health promotion by raising awareness about new studies and research. Grace described messages at the Expo as a "trigger for learning" and Mark characterized the Expo as a place to "gauge what is going on in the world and look at sort of health trends or perceived health trends." ; Additionally, knowing about available products was useful for RDNs' service providing abilities. Meredith noted, "It increases my ability to connect to people and then to provide better services." Susan explained: A majority of the time when you're working with a patient/client, you're trying to help them navigate that grocery store...So again, as dietitians we're the experts, because we say we're the experts, but we really should be the experts. And we should be helping our clients better decide on what products are valuable to their optimal life and health. (Portman, 2016)
Carreer/employement opportunities	Interactions can create business and career possibilities for NP	6	Nutrition congresses become recruitment offices for people needing a job. (Gomes, 2013)<br><br>"I wonder what clear benefit members are receiving, aside from RDNs that are employed by industry," Bellatti continues. ". (Palmer, 2015)
Source of income	Marketing partnerships/collaboration is a considerable source of income for NP	7	Both RD and non-RD bloggers had sponsored posts, providing bloggers with free products and/or financial compensation in exchange for reviewing the products. (Chan, 2020)

Code	Description	No. of documents	Select illustrative quotes
Prestige	Industry interactions can potentially confer prestige on NP (e.g., by enhancing their reputation and/or perceived influence)	2	Benefits flowing to professionals and researchers from these arrangements include financial support from a resource-rich private sector, symbolic kudos of being associated with high profile media events and a sense of being an influential political player. (Dixon, 2004)
**Advantages for the public**			
Positively influence industry (food supply and actions)	Interactions with industry allow NP and professional organizations/bodies to influence positively industry's policies, actions, and the food supply	13	A number of RDNs felt that companies were motivated to produce healthful products or, at the very least, engage meaningfully with dietitians to create a two-way exchange of information. Kate said, "A smart rep will say, well, what could we do to make it better. And then take that information back to the company." These RDNs were encouraged by the back-and-forth dialogue with both representatives. (Portman, 2016)<br><br>After this plan is approved by the Board, Academy staff will implement it. We will influence the food industry and improve the health of people in this country and around the world. (Connor, 2015)
Shape public food choices and improve public health	Interactions with industry can contribute to improving public health (e.g., by improving population dietary intakes or alleviating malnutrition)	13	As leaders in nutrition communication, food and nutrition practitioners need to continue to strengthen skills, update competencies, and document outcomes. Suggested techniques are to: • partner with industry, government, academia, and organizations to promote environments and messages that facilitate healthy food, activity, and lifestyle choices... (Freeland-Graves, 2013)<br><br>May result in increased consumer selection of products meeting standards of nutrition guidelines (Tobin, 1992)
Nutrition education and information	Partnerships/collaborations with industry can allow NP to disseminate accurate nutrition information to the public	11	Could be a forceful voice against food and nutrition misinformation.; Provide reliable nutrition information. (Ashley, 1995)<br><br>Collaboration with industry is likely to continue as opportunities arise and is viewed as being beneficial in terms of […] ensuring that information distributed is accurate, balanced and up-to-date. (Wynnes, 2003)
**Perceived and experienced risks**			
**General**	Risks that apply to NP, professional bodies/organizations and the public		
Neutralising criticism	Relationships with industry actors might result in the public, NP or their professional body refraining from criticizing industry actions, or encouraging people to engage in critical thinking around industry behaviour and actions	17	DFPI's main mission is to advocate for the Academy to sever its ties to its current Big Food partners and sponsors (including, but not limited to, Coca-Cola, PepsiCo, General Mills, and Kellogg's) since these partnerships compromise professional integrity, get in the way of sound nutrition messaging, and can inhibit public criticism of these companies' more egregious practices. (Dietitian for Professional Integrity, 2013)

Code	Description	No. of documents	Select illustrative quotes
			And in most countries now, much if not most nutrition research, especially in 'mature market economies' like North America and Western Europe, is funded at least in part by industry. People usually do not bite the hand that feeds them. (Gomes, 2013)
Being a vehicle for industry marketing and messaging	Interactions with industry are a vehicle for industry marketing that can affect NP practice and ultimately public food choices	16	Scientific events in the health sector are often supported by various sectors of industry that use them as opportunities to reach thousands of professionals and students and may therefore represent important strategic marketing actions within the context of political and economic disputes around ultra-processed food. (Canella, 2015)<br><br>In what appears to be a strategic move to further encourage women to consume alcohol, especially beer, through the NBS, a female nutritionist focused on how beer consumption improves women's health and wellbeing. (Dumbili, 2019)
Creating a positive association and credibility for industry	By establishing partnership or relationship with industry, it gives industry credibility and a positive image by association	14	The association of industry with research groups, societies, and institutions of the health sector, provides it with legitimacy and credibility; it also allows it to gain the loyalty of health professionals, who act as leaders in public opinion (Barquera, 2018)<br><br>Canadian dietitians (unwittingly) provide third party credibility in this case and according to advertising executives, ''marketing is a battle of perception … truth has no bearing on the issue [and] … third party credibility reinforces the product's positioning in multiple media appearances'' (as quoted in Rampton and Stauber, 2001, p. 58) (Gingras, 2005)
**For health policy**	Interactions with industry may influence public health policies. Those interactions might contribute to framing the debate around food and health in a way that could be favorable to industry. Interactions and collaborations can also help more directly corporation lobby efforts. Finally, it can influence public health policies by introducing industry bias in policies or dietary guidelines and programs.		
Framing the debate			
Influencing position about food products or public health messages (narrative)	Industry interactions may influence the narrative of NP and professional bodies	15	Some of the Academy's position papers employ an industry-friendly narrative. Its 'Total Diet Approach to Healthy Eating' position paper—drafted in 2013, retired on 31 December 2016 and reaffirmed to be updated, though 'still in effect' as of September 2017—states: ▶ 'All foods can fit … if consumed in moderation with appropriate portion size and combined with physical activity.' ▶ 'Classification of specific foods as good or bad is overly simplistic and can foster unhealthy eating behaviors.' ▶ 'Focusing on variety, moderation, and proportionality in the context of a healthy lifestyle, rather than targeting specific nutrients or foods, can help reduce consumer confusion'. (Bellatti, 2019)<br><br>In any case, the message of the intervention was well received by the students I was able to interview since most of the latter remembered that "breakfast

Code	Description	No. of documents	Select illustrative quotes
			cereals were good". The very large majority of the students interviewed did not take this intervention at all as being advertising, […] (Scheffer, 2015)
Influencing professional body/health organization's policy position(s)	Relationships between industry actors and professional bodies/health organizations can influence policy positions of the latter	5	Does Sponsorship Impact AND Lobbying and Policy Positions? To its credit, AND has a political action committee, ANDPAC. More health groups should have PACs to be an effective political player. However, AND's policy positions remain mostly self-serving, relatively safe and noncontroversial. While there is no way to know for sure if the corporate sponsorships are playing a role, it's disturbingly clear the group is not showing leadership on the most pressing nutrition policy issues of our day. (Simon, 2013)<br><br>Almost a quarter of the speakers at the 2012 AND conference, attended by up to 10,000 dietitians and other health professionals, had industry ties. AND does not support limits on soft drink sizes, nor soft drink ('soda') taxes. (Gomes, 2013)
Introducing industry bias in public policies and programs	Interactions with industry may influence nutrition/public health policies and programs	5	Although the Congress included a wide variety of institutions and approaches, this situation typifies the infiltration of commercial interests into scientific discussions (Monteiro, 2017) and the resulting risk of industry bias in public policies and dietary recommendations. This influence was clear in the narratives in the flyers about the products exhibited at the commercial stands and the relationship with the themes and presentations in sponsored symposia and other scientific activities. Three thematic axes were identified to provide a health halo: fortification with micro-nutrients, reformulation of products, and symbolic reconversion of snacks. (Piaggio, 2020)<br><br>This study found evidence that major Australian food industry actors engage in diverse and extensive practices which can have an influence on public health policies and programs. (Mialon, 2016)
Favouring industry lobbying	Interactions may facilitate/support corporate lobby efforts and ultimately risk undermining public health policy (e.g.: delay, neutralize, etc.)	3	Soda companies also can neutralize potential legislative opposition by invoking reciprocity and financial dependence on the part of national health organizations. (Aaron, 2017)<br><br>Partnerships also help with corporate lobby efforts. For instance, Coca-Cola CEO Sandy Douglas leveraged the company' s relationship with the American Academy of Family Physicians to help make the case that soda taxes were unnecessary. Save the Children, an organization aiming to positively change the lives of children, was initially a staunch supporter of soda taxes. Recently, the organization withdrew its support, saying that support of the soda taxes did not fit the way Save the Children works. (Freedhoff, 2011)
**For professionals and organizations**			
**Image / Reputational risks**			

Code	Description	No. of documents	Select illustrative quotes
Trust, credibility, integrity, and reputation	Interactions could put in peril public trust, credibility, integrity, and reputation. Both for professionals and organizations	32	I'm a positive RD, but think the photo ban by @eatrightFNCE #FNCE violates 2 things consumers crave most: transparency and trust. --- @Greeneating (Dietitians for Professional Integrity, 2013)<br><br>Our credibility is contingent on our trustworthiness. If we cannot be trusted, our raison d'être is compromised. (Gingras, 2005)<br>"It's a really bad view; it undermines the perception of the integrity of the profession when there are industry groups clearly contributing financially to a [scientific] conference, or even just being there." [Academic] (Mialon, 2017)<br><br>However, 18% of members would like the BDA to have complete independence from corporate partners, sponsors, and advertising: 9 members claim that due to commercial collaborations the BDA is losing its credibility and that there is reputational damage. (British Dietetic Association, 2017)
Appearance of endorsement, impropriety and/or commercial bias	Relationships or interactions with industry might be a risk of giving an appearance of endorsement of a specific product or industry by a NP/professional body. It can also give the appearance of impropriety or commercial bias from the public's or other health professionals' perspectives	8	Although alliances do not necessarily imply an endorsement of the partner's products, they may well give the appearance of doing so (Nestle, 2001)<br><br>May construe message on a product as a direct product endorsement by professional organization (Tobin, 1992)<br><br>Because nutrition and dietetics practitioners utilize evidence-based research in their practice when making nutrition-related recommendations, the receipt or solicitation of funds can give the appearance of impropriety. (Boyce, 2017)<br><br>Although sponsorship of journals and conferences may not directly influence editorial content or the opinions of conference speakers, it may well do so in more subtle ways or give the appearance of doing so. (Nestle, 2001)
**Compromising independence**	NP and organizations' objectivity and judgement can be compromised by interactions with industry		
Impairing objectivity and judgement	Relationship with industry may put in peril NP independence, objectivity and professional jugdement	19	Thus, dietitians are in a conflict of interest since Xenical causes their clients to experience deleterious nutrition status (prevents absorption of fat soluble vitamins, intensifies intestinal evacuation, heightens dietary fat phobia), yet the dietitian benefits when physicians refer the same clients to seek the services of said dietitians. The dietitians' interest in ongoing referrals from physicians interferes with her/his clinical nutrition judgment. (Gingras, 2005)<br><br>Sponsorship of scientific societies and foundations active in the field of childhood nutrition and obesity, by an industry that markets ultra-processed foods and sugar-sweetened beverages causes a conflict of interests among the professionals of these organisations,8 with the ensuing risk that their professional judgement or actions may be unduly influenced by non-health-related interests, due to feelings of gratitude or reciprocity, which may be

Code	Description	No. of documents	Select illustrative quotes
			unconsciously induced by even the smallest gifts. (Guzamn-Caro, 2020)
Influencing decision-making and recommendations	Recommendations and health messages may be at risk of being biased or influenced by interactions between NP and industry	17	Industries seek to induce the recommendation, the prescription and the consumption of products by students and nutritionists through strategies such as sponsorship of scientific meetings, travel funding and the distribution of promotional gifts. However, acceptance of these gifts can generate a moral obligation to reciprocate, thereby jeopardizing the judgment of information and decision on professional conduct. (Pereira, 2016)<br><br>Corporate dollars always introduce perceived or real biases that may taint or distort evidence-based lifestyle recommendations and health messages. (Freedhoff, 2011)
Contradicting organization and professional's public health mission	Interactions may contradict the organization's mission	5	Many RDs felt that the Academy's current sponsors contradicted the organization's mission. One member bluntly stated, "Right now we think the sponsors that we have are not promoting the Academy's mission, and that's a big problem." Another RD voiced concerns about being sponsored by food companies that sell processed foods. She felt that "it's kind of conflicting with our mission of being a nutrition expert, because a nutrition expert probably won't recommend very many of those foods.". (Smith, 2014)<br><br>The authors also singled out AND's partnership with Hershey's as a notable conflict with the group's vision "to optimize the nation's health through food and nutrition:" When the Hershey Center for Health & Nutrition became a partner of the Academy to collaborate on their new campaign titled "Moderation Nation," the announcement stated that they would be working with the Academy to provide resources for the American public to achieve a healthy balance in terms of food (including sweets) and exercise. (Simon, 2015)
Losing members	NP might give up its membership or not seek membership of an organization if they do not agree with organization's interactions with industry	1	The Academy of Nutrition and Dietetics' recent history of food industry ties has resulted in some members publishing 'statements of concern',. These dietitian-penned accounts shed light on why some dietitians renounced their memberships. Many of these first-person pieces call for sponsorship reform and question whether the Academy's actions align with its mission ('a world where all people thrive through the transformative power of food and nutrition') and vision ('accelerate improvements in global health and well-being through food and nutrition'). (Bellatti, 2019)
**For professionals**			
**Negatively impact content of NP education**	NP education might be negatively impacted by industry involvement in it by creating bias in continuing education, influencing student and teaching programs, or by influencing the scientific content of nutrition events/conferences		
Influencing scientific content of an event	Industry interactions can influence the content of conferences/events, including materials provided	7	An example of industry influence on the content of events was the cancellation of a debate on childhood obesity during the 16th World

Code	Description	No. of documents	Select illustrative quotes
			Congress of Food Science and Technology (August 5–9, 2012, Foz do Iguaçu, Paraná State, Brazil) after the president of the meeting argued that the issue "would cause inconvenience to potential sponsors"[12]. (Canella, 2015)<br><br>One of the criticisms of corporate sponsorship is how it might impact dietitians' education. "We see examples at our conference of educational materials that repeat industry talking points disguised as science," Bellatti says. (Palmer, 2015)
Influencing continuing education content	Continuing education can be biased by industry interactions/collaborations	4	Other interactions may affect the practice of nutrition professionals, and nutrition education—not just continuing professional education of nutritionists and dieticians but, as the AAFP–Coca-Cola example demonstrates, public education and information. (Marks, 2011)<br><br>While certain continuing education programs may have a strong level of credibility, in my opinion there are unfortunately many that come off sounding like commercials or endorsements, and it's hard for RDs to receive an unvarnished, multiperspective update of the latest science from food companies and their sponsored materials.". (Palmer,2015)
Influencing students' training/teaching programs and careers	Industry interactions can influence educational content of training programs and NP's careers	1	These actions are essential for students to receive reliable information, based on reliable data and independent of commercial interests, so that trained so that the trained nutritionists can perform their with ethics and autonomy, promoting better conditions for the conditions for the choice of an adequate and healthy and healthy food, considering that human food is not only related to biological needs, but is impregnated by the culture and society that surrounds it. the society that surrounds it. (Pereira, 2016)
Unconscious influence on NP practice/beliefs	Interactions can constitute a risk of industry influence/bias when professional are not conscious of this potential influence and do not recognize it, or when the influence is done in a subtle way	7	The power imbalance in many nutrition partnership initiatives begs a question regarding the risks of professional and scientific capture. Their very ubiquity makes them appear benign and unworthy of scrutiny. (Dixon, 2004)<br><br>[S]ponsorship generate conflict of interest, which introduces a subconscious bias in favor or the donor company. (Aaron, 2017)
Creating antagonism between professionals	Interactions with industry may influence NP's position about public health nutrition advice which might create antagonism between professionals	2	One participant mentioned the role of nutrition professionals working in the food industry who present their views in the media: "There are even some of those nutrition professionals who do work with the food industry becoming quite vocal in the media about [a recent public health program under development] as well, bringing that industry-flavored position into the public arena, which really is unhelpful from a perspective of trying to give the community clear and unbiased advice, when we, as a nutrition community, appear to be debating among ourselves, where some people in the nutrition community are actually coming from a particular industry standpoint."

Code	Description	No. of documents	Select illustrative quotes
			[Senior manager at a public health-related not-for-profit organisation] (Mialon, 2017)
Misleading information/messaging and resulting confusion	Interactions or involvement of industry in research and professional education might be misleading for NPs and create confusion	2	We [authors, Dixon et al] contend that nutrition and dietetics organizations willingly participate in ventures with the food industry, even though it is apparent that many such alliances confuse product sponsorship and health promotion. This contention is supported indirectly by Nestle [(Nestle, 2002), p. 21], when she notes how the food industry and the nutrition profession currently speak with one voice when they intone 'there is no such thing as a bad food' and 'eat a balanced diet'. (Dixon, 2004)
Revocation of the licensure	Improper interactions with industry can result in NP losing their license/accreditation	1	Revocation of licensure is a possible ramification. (Unknown, 2000)
**For the public**			
Undermine public health nutrition recommendations	Interactions with industry may undermine public health recommendations and ultimately public health	20	According to these RDs, corporate sponsorship allows the food industry to promote their products through the Academy, negatively impacting public health. (Smith, 2014)<br><br>Although strategic alliances are needed to solve problems associated with malnutrition, I recognize the obstacles that can arise from engaging in relationships with actors who have potentially different primary interests; that is, interests that might infringe on health or common wellbeing (children's rights, right to food, wellbeing, right to information, and right to water).[55,56] (Barquera, 2020)
Misleading information and resulting confusion	Interactions can mislead the public and create confusion around food and health. Health messages may not be coherent or can be contradictory; they can also be biased by the omission of important information which can mislead consumers/the public	15	Nutrition and dietetics practitioners who accept payment to promote a product or service must disclose this marketing relationship. Otherwise, it is misleading to the consumer who is unaware of the conflict of interest. (Helm, 2016)<br><br>Some professional associations continue to accept fees to endorse sugary breakfast cereals and processed snack foods, even though this practice was considered potentially deceptive by state attorneys general nearly a decade ago. (Ludwig, 2008)
Increasing price of endorsed products	Interactions resulting on perceived/actual NP endorsement of industry products may increase prices of such products	1	Price of product may be increased (or overemphasize single category of not so low as it could be) due to costs of endorsement (Tobin, 1992)
