# Supplementary material for: Interactions Between Nutrition Professionals and Industry: A Scoping Review
Source: Int J Health Policy Manag. 2023 Aug 22;12:7626. doi: 10.34172/ijhpm.2023.7626 (PMC10590255; doi:10.34172/ijhpm.2023.7626)
Supplement: Supplementary file 5 — Codebook With Illustrative Quotes – Strategies and Actions Proposed or Used, to Address and Manage the Risks Associated With Nutrition Professionals and Industry Interactions. [file ijhpm-12-7626-s005.pdf]

Article title: Interactions Between Nutrition Professionals and Industry: A Scoping Review

Journal name: International Journal of Health Policy and Management (IJHPM)

Authors' information: Virginie Hamel[1,2]*, Marita Hennessy[3], Mélissa Mialon[4], Jean-Claude Moubarac[1,2]

[1]Department of Nutrition, Faculty of Medicine, University of Montreal, Montreal, QC, Canada.

[2]Centre de recherche en Santé publique, Montreal, QC, Canada.

[3]College of Medicine and Health, University College Cork, Cork, Ireland.

[4]Trinity Business School, Trinity College Dublin, Dublin, Ireland.

***Correspondence to**: Virginie Hamel, Email: virginie.hamel.1@umontreal.ca

Citation: Hamel V, Hennessy M, Mialon M, Moubarac JC. Interactions between nutrition professionals and industry: a scoping review. Int J Health Policy Manag. 2023;12:7626. doi:10.34172/ijhpm.2023.7626

Supplementary file 5. Codebook With Illustrative Quotes – Strategies and Actions Proposed or Used, to Address and Manage the Risks Associated With Nutrition Professionals and Industry Interactions

Code	Description	No. of documents	Select illustrative quotes
**Strategies and Actions, Proposed or Used, to Address and Manage the Risks Associated with Nutrition Professionals and Industry Interactions**			
**1) Institution level/organization/professional body level**	Strategies that can be used by professional body, nutrition and health organizations and educational institution to managed or limit risks associated with interactions between industry and NP		
**Management**			
**1,1) Codes, policies and guidelines**			
Codes, policies, guidelines, etc.	Institution should use and consult codes of ethics, guidelines, or policy to help managing industry interactions. This includes the recommendation of developing these codes, guidelines or policies if not done yet, and the revision of existing codes, where necessary	32	"I agree with DFPI that the problem at hand is not collaboration with the private sector, but rather who in the private sector we align with. For this reason it is critical to propose regulations and guidelines around partnerships in order to preserve the integrity of the RD credential. (Dietitians for Professional Integrity 2013)<br><br>SLAN's position on the problem CDI is summarized in the following 12 guidelines basic, with which it is intended to prevent it and establish mechanisms to manage it. The Latin American Nutrition Society:<br>[…] (Barquera, 2018)

Code	Description	No. of documents	Select illustrative quotes
			Bellatti believes the Academy would benefit from very specific guidelines about what responsible, ethical, appropriate, and relevant sponsorship for a nutrition organization should look like. "Right now the guidelines are vague and completely open to interpretation," he says. "The Academy could act much like the World Health Organization and publicly denounce the food industries that battle public health policy and get in the way of health.". (Palmer, 2015)
Monitoring and evaluation of the respect of codes, guidelines, and policies	Implementation of codes and guidelines should be monitored and evaluated	4	Guidelines need to be agreed, and compliance with these monitored. (Margetts, 2009)<br><br>Like any other public policy, rules of engagement with industry need to be monitored and evaluated regularly. In our profession this should be part of the responsibility of the IUNS and its constituent bodies, and also of the United Nations System Standing Committee on Nutrition (UN SCN). The results of such appraisal need to be published regularly in an appropriate journal, such as Public Health Nutrition. Monitoring and evaluation needs to include maintaining of records and of signed forms of conflicts of interest, and of all relevant reports. (Oshaug, 2009)
Sanction when non-compliance observed	Members of institution should be sanctioned in cases of non-compliance	1	The implementation of these policies is as much important as their development, and sanctions could be considered in cases of non-compliance. (Mialon, 2020b)
Having or develop more selective criteria for choosing sponsors	Institution should have agreed criteria for choosing sponsors (e.g., for a conferences/events or for the organization itself)	7	Scientific entities and their organizing committees for events should consider whether their goals are compatible with the objectives of the food and beverage industry. Key questions that should be posed include: 1) “Are there any alternative sources of support that would not generate conflicts of interest in scientific events?”; 2) “What potential interests are driving the participation of food and beverage industries in the events?”; and 3) “Have conflicts of interest emerged in other events or scientific spheres as a result of the participation of the prospective sponsors?” (Canella, 2015)<br><br>How are partnership decisions made? Corporations and other organisations must meet well-defined criteria before they join forces with ADA and the ADAF. Groups must have a commitment to market products and or services that conform to ADA’s official positions, agree with ADA’s key messages on relevant issues, and support our key education philosophies: There are no good or bad foods (Derelina, 1995)
Dissemination of codes or guidelines to mitigate or manage COI	Dissemination of code of ethics or guidelines to members to ensure principles that guide NP conduct in respect to industry interactions would be known and accessible	4	Another felt that the rules regulating corporate sponsorships need “to be much more definitive, much more transparent, and much more publicly accessible.” (Smith, 2014)<br><br>Among the strategies to achieve the above the code on CDI and its guidelines should be disseminated to ensure members are informed about the problem and the rules of the SLAN about it. (Barquera, 2018)
Invest in human resources to assist with COI management	In their financial management, it is recommended that organizations should invest in more human resources that would assist with COI management	1	"Where I do see room for improvement is with affiliates and DPGs that have fewer human resources to manage the oversight and policy enforcement related to sponsorship of continuing education programs," (Palmer, 2015)
**1,2) External or internal consultation**			
Consider members’ opinion regarding institutional sponsorship	When institutions are looking for sponsorship, they should survey members to help decision-making	5	How about letting members vote on sponsors or partnering with tech companies instead of food companies. (Palmer, 2015)

Code	Description	No. of documents	Select illustrative quotes
			Additionally, most respondents agreed that members should have a say in deciding who should be Academy sponsors (83%) (Reitschamer, 2012)
Creating a conflict of interest/ethics committee	Creation of a COI or ethic committee within the institution to consult when necessary	7	Due to the urgent need for studying and managing relationships between the food and beverage industry and the field of health and nutrition research, in 2017, the President of the Latin American Society of Nutrition (SLAN) appointed a Conflict-of-Interest Committee (CCI). The CCI was charged with the development of a proposal of a position for the management of conflict of interest (COI) for consideration by SLAN. This document details the work of CCI, and the position adopted by SLAN. (Barquera, 2018)<br><br>We also emphasize the need [for] […] the creation of committees that analyze the CoI (Pereira, 2016)
Independent advisory group to evaluate sponsorship activities	Organization could consult regarding proposed sponsors with an independent advisory group outside the institution (exact details were not specified)	3	Key to the second alternative are management approaches intended to minimize bias and enhance transparency, such as 1) the establishment of an independent advisory group reporting directly to the ASN board and charged with reviewing proposed activities cosponsored by entities and/or individuals at interest (Garza, 2019)<br><br>Noting that there is little agreement on what is right or wrong in these situations, the agency recommended that institutions form committees with independent members outside the institution, collect information on financial relationships, conduct educational programmes, review agreements made by individual investigators, and manage their own conflicts of interest64. Although compliance may continue to pose problems, these suggestions encourage attention to the issues and deserve to be taken seriously. (Nestle, 2001)
**1,3) Alternative financial strategies**	To minimize interactions with industry, alternative financial strategies to decrease the reliance on the industry's financial resources were proposed		
Seek alternative/non-conflicted sponsorship or funding	When looking for sponsor, organizations should seek alternative/non-conflicted sponsors (i.e., sponsors that do not conflict with their mission and public health objectives, such as sustainability, environmental companies or companies different from UPF industry)	10	Another felt that "there are plenty of companies out there that would probably be very happy to benefit from getting the word out about their products—event if it's not food products, if it's technology companies, sustainability, environmental companies, anything really that doesn't have to do with junk food." One member felt that the problem would be solved when the assortment of corporate sponsors has "a better balance…so it's not so heavy on the all the junk food." (Smith, 2014)<br><br>As shown by the Academy's 2012 annual report, only 6% of revenues come from corporate sponsorships (AND annual report, 2012). However, most HEN members could not imagine a scenario in which the Academy could exist without their sponsors. While the majority of HEN members' offered reformative solutions, some RDs suggested transformative solutions. For example, one member felt like the recently passed Affordable Care Act could offer more government funding to replace corporate sponsorship. She hoped the Academy would partner with healthcare agencies to "[take] advantage of some of the funding that coming out that's supposed to be supporting prevention." (Smith, 2014)
Cut down expenses and revise priorities	Review organization's budget to identify areas of possible savings and propose changes in events and congress	7	But do conferences really need additional money from industry to break even? Or do such funds mainly go towards supporting five-star hotel lifestyles for the organizers and

Code	Description	No. of documents	Select illustrative quotes
	structure to cut down expenses and rely less on corporate sponsorship (e.g., holding events online or replace prestigious/expensive venues and locations with more simple or less costly options like university)		prestigious speakers? Further, if money and support is needed from industry, why does this have to come from sectors of industry whose profits depend on products whose regular consumption often is harmful to public health? Why do organisers evidently not seek support from say the transport or insurance industry? (Oshaug, 2009)<br><br>Evaluate the Academy's budget to: • Identify areas of potential savings that could help offset possible future changes in sponsorship. (Dietitians for Professional Integrity, 2013)
Increase membership fee revenue	Members could pay more for their membership as a goal to reduce the need for industry sponsorship	2	Evaluate the Academy's budget to : […] • determine likely cost(s) members would need to absorb with possible future changes in sponsorship, and poll membership on willingness to absorb said costs (with specific figures given). (Dietitians for Professional Integrity, 2013)
**1,4) Compromise and other strategies**	Strategies that allow certain interactions by compromising to minimize risks and COI and other strategies		
Structural changes at conferences/events	Introduce structural changes such as putting exhibits in another room apart from the conference/ event and to stop offering a bag with educational materials	4	DFPI sets forth the following recommendations for future FNCEs: 1. Remove the ban on expo floor photography. If some exhibitors are concerned with negative press or public outcry, they should consider more carefully the messaging displayed at their booths. 2. Only allow conference-related information in the FNCE tote bag (i.e.: a listing of sessions and a map of the expo floor), not materials written by industry. 3. Seek out and invite more advocacy groups like Corporate Accountability International, The Union of Concerned Scientists, The Environmental Working Group, and Food Chain Workers' Alliance to the expo. No funding or any other support is acceptable from entities identified here as Big Food, other than exhibition stands located in a separate hall, of which the revenue goes to independent public health program. (Dietitians for Professional Integrity, 2013).<br><br>This special arrangement was presented by the organizing committee as a way to differentiate the commercial aspect from the scientific one during the Congress experience. This was explained on a poster next to the entrance: "Following the directives of the ethics committee, this space has been designated for commercial activity exclusively". (Piaggio, 2020)
Accept less risky interactions	Only accept interactions that are considered acceptable (e.g., accepting contributions from industry in the form of travel, meals, honoraria and conference while refusing contribution to educational or research programs)	2	Another compromise is to argue that industry contributions in the form of travel, meals, honoraria, and conference sessions pose minimal problems but that the potential for conflicts increases when companies contribute to educational or research programmes. (Nestle, 2013)
Ensure the educational materials used are free of explicit or implicit bias	When establishing partnerships with industry for educational material, care should be taken to ensure that the materials are free of bias	1	In general, educational, and informational materials, whether produced by a professional society and funded by industry, produced by industry and endorsed by a professional society, or produced jointly by both, offer the possibility of wide distribution of responsible and accurate information that is also specific and useful. However, great care should be taken to ensure that the materials are free of either explicit or implicit bias and that no general approval of sponsoring industries' products is implied. (Tobin, 1992)
Dilution principle	Organizations could engage more sponsors to dilute the influence of single actors	2	Several respondents noted that they felt that additional sponsors would dilute the effect of any one in particular and about half simply said that the more money that could be gained from corporations to be used by the Academy, the better. (Reitshamer, 2012)

Code	Description	No. of documents	Select illustrative quotes
			8 members suggest that the BDA needs to work with a wider range of parties. (PARN, 2017)
**Education**			
**1,5) Educate NP about industry interactions and related COI**	Educate NP about industry interactions and related COI	10	DFPI also aims to educate RDs, other health professionals, and the general public on the general issue of problematic partnerships between Big Food and the public sector (i.e.: health organizations, federal health campaigns, food and nutrition non-profits). (Dietitian for Profressional Integrity, 2013)<br><br>We also emphasize the need to include critical content in the course menus and and pedagogical projects of educational institutions about the performance of the food industry to influence health professionals; the promotion of debates about CoI with the participation of and researchers. (Pereira, 2016)
**1,6) Strategies to favor independence in educational settings**			
Independent accreditation of university dietetic training	Assure independent accreditation of university dietetics training separate from professional bodies with potential conflict of interests	1	At minimum, Australian dietitians need to be given the choice to be professionally registered with a body external to the DAA. Similarly, accreditation of Australian University dietetic training needs to be completely separate from the DAA while the conflict of interest exists.59 (Simon, 2015)
Present variety of products instead of a particular brand	A variety of products could be discussed/presented in nutrition educational institution setting, instead of a single-branded product	1	Educational Programs: Colleges and Universities How can educators strive to avoid duality of interests? In the college and university setting, they can present a fair and balanced description of a variety of products rather than focus on the ones manufactured or sold by only one or two companies. (Unknown, 2005)
Use independent documentation, references, and teaching materials	Education settings for future NP should use documentation, references, and teaching materials independent from industry brand or products	1	As they describe the results of research, they can explain the limitations of studies and teach students how to properly assess sampling techniques, methods, and results. They can also carefully screen teaching materials and reference lists; and, as necessary, they should exclude those that are biased in favor of any specific product. (Unknown, 2005)
**2) Individual level**			
**Management**	Strategy to deal or manage current or future interactions with industry		
**2,1) Tools or resources for decision-making**	Tools or resources are suggested to help decision-making about CoI or to evaluate if corrective actions have to be undertaken		
Code of ethics and guidelines from professional body/association/organization	Code of ethics and guidelines (e.g., guidelines for sharing information on social media) from professional bodies/organizations are tools that can help NP to manage interactions with industry and related COI	16	The following principles of the "Code of Ethics for the Profession of Dietetics"3 are particularly relevant for dietitians in business: 1. The dietetic practitioner provides pro-fessional services with objectivity and with respect for the unique needs and values of individuals. The dietitian is called on to be client oriented instead of self-oriented. 4. The dietetic practitioner conducts her self/himself with honesty, integrity, and fairness. This professional standard should take precedence over business practice and behavior; if the two are in conflict, ADA 's standard should be held in higher regard. 5. The dietetic practitioner remains free of conflict of interest while fulfilling the objectives and

Code	Description	No. of documents	Select illustrative quotes
			maintaining the integrity of the dietetic profession. […]. 12. The dietetic practitioner promotes or endorses products in a manner that is neither false nor misleading. […] The following hypothetical situation offers one example of how ADA's Code of Ethics can be used to make business decisions about a nutrition product. (Hermann, 1992)<br><br>Dispensing supplementation advice in the office or on the Internet and marketing supplements out of a private practice office have become available as additional sources of income. But before proceeding with any plans to take advantage of these opportunities, urges Lisa K. Fieber, MS, RD, Chair of the Nutrition in Complementary Care dietetic practice group, you should familiarize yourself with federal, state, and local laws as well as The American Dietetic Association (ADA) Code of Ethics. (Fieber, 2000)
Other tools	Principles to guide NP in managing COI (DORM for **D**isclosure/ **O**ptions/ **R**eassurance/ **M**odification and Nolan principles)	2	The DORM Principle In circumstances where an RD may be permitted to practice while in a conflict of interest, the College advises that RDs be as transparent as possible. Certain situations can be managed using the safeguards outlined in the DORM Principle: Disclosure: at the earliest opportunity, RDs should disclose the nature of the conflict to the client; Options: inform the client of his/her alternatives and assist in arranging for alternatives where requested; Reassurance: reassure clients that choosing another product or service will not affect the quality of the professional services to them; Modification: making small modifications can remove or greatly reduce the potential for conflict of interest. (Cohen, 2009)<br><br>Requests for endorsement of products by registered dieticians are an ethical dilemma. Sound scientific justification is always needed when endorsement is required which can be guided by the aforementioned principles of Nolan. (Kunneke, 2018)
**2,2) Individual discernement**	Critical thinking and professional judgement can be used to solve ethical problem link to industry interactions		
Balance risks and benefits on a case-by-case basis	To help with decision-making to manage a COI, NP can balance risks and benefits on a case-by-case basis (e.g., in context of corporate funding or accepting gift/ payment)	4	Should researchers, practitioners, professional societies, and academic departments `just say no' to corporate funding62? It is unrealistic to think that doing so will become common practice, not least because of the evident benefits of such arrangements. Given current funding realities, the most useful approach may be to balance risks and benefits on a case-by-case basis. (Nestle, 2001)
Fact and references checking and follow up	Check facts and references found in information or educational material made by industry. NP should also base their practice on independent research	3	Lastly, RDNs spoke of the need to do additional fact checking and follow up on the information gleaned at the Expo. Susan noted “you’ve definitely got to go back and do more homework” and Anne said, “if it’s referenced, I would certainly go to the references and see how the study was done. ”The current landscape of Academy/industry relationships has resulted in the utilization of three specific strategies on the Expo floor: avoidance, professional discernment and additional follow up. (Portman, 2016)<br><br>Dietitians should also be cautious when they recommended the purchase of certain food products and supplements for patient care. This should be based on the individual nutrient needs of patients and not a specific brand name for a product they happened to see advertised or heard about at a conference. While making such recommendations, dietitians should be prepared to provide documentation such as research that is

Code	Description	No. of documents	Select illustrative quotes
			conducted by an independent party and not supported by the company that manufactures or distributes the product they are suggesting (Unknown, 2005)
Rely on professional judgement	Professional should use their professional judgement when they evaluate the validity of the message and information they received from industry and how they use it in their professional practice	2	[…] or a number of RDNs, instead of avoiding messages altogether, they relied upon their professional judgment to assess the claims that were being made on the floor. Professional judgment relied upon both formal training and professional experience to evaluate the validity of messages at the Expo. As experts in nutritional health, these RDNs felt confident in their ability to intrinsically discern fact from fiction and health promotion from commercial sales. (Portman 2016)<br><br>Principle 19a states: “The dietetics practitioner does not engage in dishonest, misleading, or inappropriate business practices that demonstrate a disregard for the rights or interests of others”: However, these principles are further clarified within the code to explain that whether a gift or incentive is viewed as improper depends on all factors relating to the transaction, and this does not mean that nutrition and dietetics practitioners cannot serve as consultants or participate in events, such as conference, where meals and promotional samples are shared. Knowing how the ‘when’ and ‘where’ affect the ‘why’ is a matter of judgement and the topic of many questions within the field, Polly and Dierks said. (Boyce, 2017)
**2,3) External consultation and advice on COI**	NP and students can seek external advice, including from colleagues or expert organizations, when they face a situation of COI	5	“Discussing the situation with a colleague is often a very good approach,” says Papoutsakis. “Speaking with other people in my field to see how others perceive the situation has always been very helpful to me.” Researchers in the field ofhealth care ethics suggest reaching out to a mentor or a member of your dietetic practice group to get additional perspectives on the potential COI, as well as approaching leadership in your facility or organization to get an administrative, policybased view of the situation. (Peregrin, 2020)<br><br>Both Dierks and Polly said more information is good and ongoing discussions among colleagues is ideal in helping stave off ethical conflicts between individuals and the organizations with whom they work. (Boyce, 2017)
**2,4) Accept industry funding through third party**	Accept industry funding through third party, not directly from industry	1	Because I do not accept honoraria from food companies, I had P&G write the checks to my department's scholarship fund or to CSPI.(Nestle, 1998)
**2,5) Retain control over the content**	NP should make sure that they retain control over the content of the message when establishing a partnership with industry	1	Dietetics practitioners can ethically act as consultants and speak on behalf of a company or product as long as they retain control over the content and disclose their relationship with the company. (Woteki, 2006)
**2,6) Document management of COI**	Document COI and how it has been managed	1	It is important to document any real, potential, or perceived conflict of interest and how it was managed. If the conflict of interest was managed via the DORM Principle or otherwise, clear documentation of the disclosure, options, reassurance and modification (as applicable) should be included (Cohen, 2009)
**3) Institution/ organization/professional body and individual level**			
**3,1) Prohibiting, avoiding, and refusing interactions with industry**	Prohibiting, avoiding, refusing, or getting rid of interactions with industry. Including rejection or avoidance of interactions or industry. Some suggest avoiding some type of interactions or industry, while other suggest avoiding all type of interactions.		

Code	Description	No. of documents	Select illustrative quotes
Individual level	Prohibition strategies recommended for individual NP including avoiding and prohibiting interactions with industry	9	However, there are certain situations that RDs should avoid entirely: - Accepting a gift, rebate, credit or other benefit for referring a client to any other service or program - Offering, making or conferring a rebate, gift, credit or other benefit for receiving a client referral from another professional - Pressuring/bullying clients to participate in research that directly benefits the RD (including monetary benefit or status) - Selecting a foodservice supplier on the basis that the supplier offered a substantial gift, or other personal benefit to an RD (i.e., paying for an RD to attend an overseas conference) - Entering into any lease or use of premises or equipment arrangements under which the amount payable by or to an RD or related person corresponds to the amount of fees charged by the RD, or to the volume of clients seen by the RD. (Cohen, 2009)<br><br>Suggested that to avoid undue influence, nutritionists should refuse sponsorship or decine invitations to attend or speak at sponsored meetings. Perhaps so, but if we take this ethical high road, we end up only talking to ourselves. (Nestle, 2013)
Institution level	Prohibiting and refusing all interactions with industry	14	Public health organizations' role is to educate so professional health associations must avoid partnerships, product endorsement fees, or other financial ties with industry that compromise their independence and public credibility. Advocacy groups should broker industry agreements only with broad-based support from the public health community. (Ludwig, 2008)<br><br>Two other interviewees noted that scientific conferences could be held independently, without sponsorship from the industry, which is already the case for the annual Congress of the Chilean Society of Family Medicine [30], for example. (Mialon, 2020, I had never)
Both professional and organization level	Prohibition strategies recommended for individual professionals and organization	6	These ties reflect poorly on our credential, because anybody who wants to criticize dietitians can make such an easy argument. If we got rid of these ties, we would take away a huge source of criticism," Bellatti says. (Palmer, 2015)
**3,2) Transparency and disclosure**	Disclosing interactions with industry and related COI		
**Institution level**			
Transparency vis-à-vis the public and members	Disclosure of institution interactions with industry and related COI	12	8 members feel that further transparency is required regarding the nature of collaborative partnership(s) and/or sponsors (British dietetic Association, 2017)
Transparency in conference and nutrition and scientific event	Disclosure of industry involvement in conference and scientific event and COI	10	Organizers of conferences should publish a statement concerning the content of their conferences, and their position on entities with interests that do or may conflict or compete with nutrition policy and practice (Gomes, 2013)
Signed agreement or contract with industry to manage/guide interactions around conference sponsorship or general sponsorship	There should be a signed agreement or contract to make clear the terms around the benefits and the content of conferences that are sponsored by industry or general sponsorship of an entire organization	3	Harriet Kuhnlein also says, 'there is good information and good intention for good partnerships without conflict of interest'. She recommends that 'there should be no conflict of interest or coercion of speakers by the sponsor. There should be a signed agreement of benefits shared with speakers, and this should be declared at the presentation and even in the programme, as in journals' (Gomes, 2013)<br><br>DAA's CEO Clare Hewat says their binding legal contracts protect DAA's independence, but how can we know that is true without seeing these documents?

Code	Description	No. of documents	Select illustrative quotes
			"DAA can and does express its opinion without fear or favour and a review of our media releases, public comments and submissions bears that out," she said.[5] (Simon, 2015a)<br><br>To avoid the public health risks of these conflicts of interest, it would be best not to accept sponsorships from industries which market unhealthy products and whose interests clash with those of public health, and instead publicly declare the sums received under and the conditions stipulated in the funding agreements concluded with the industry. (Guzman-Caro, 2020)
**Individual level**			
Transparency vis-à-vis the clients and the public	NP should be transparent with clients and the public about having industry ties and COI	11	You could be working for a food company and at the same time be working part-time for a health care organization. The challenge is to separate those two roles so that you don't let the interest of one of your employers inappropriately influence the responsibilities of your other job. You have to make it very clear to your patients and clients that you are also working for a specific food company or industry, so that if you're recommending that food or nutrient your clients are aware of your unique perspective and that you know a lot about that product, but you also might be doing it from the standpoint that you're getting paid by them. (Peregrin, 2020)
**3,3) Identification and awarness-raising**			
Advocating for COI recognition and for action by institution	Members of institution advocating for recognition of COI that occur from industry interactions	4	As far as DFPI's continuing mission is concerned, we were grateful to meet with current Academy President Dr. Glenna McCollum and Past President Dr. Ethan Bergman for 15 minutes on the last day of the conference. At that meeting, DFPI submitted its Change.org petition (which called for the Academy to cut ties with its Big Food sponsors and garnered just over 25,000 signatures) and expressed its desire to engage in dialogue with Academy leadership over this issue. DFPI is well aware that systemic change takes time, effort, and dialogue. We believe it is crucial to have ongoing conversations about this topic with Academy leadership and have incremental goals in place, with the hopeful end result of the Academy severing partnerships and sponsorships with Big Food. (Dietitian for Professional Integrity, 2013)
Identifying COI	NP should reflect about their interactions with industry to identify any COI; this can include the use of frameworks and tests	4	IDENTIFYING A CONFLICT OF INTEREST 1. The Personal Interest Test - Am I receiving any personal benefit? The RD has a personal interest when he/she receives any personal benefit from an action or decision made with respect to clients. Benefits can include, but are not limited to, gifts, advantages, discounts, status, rebate, credit and preferential treatment. (Cohen, 2009)<br>CoI Framework :<br>Using the Conflict-of-Interest Framework, the first step is to determine if anyone is relying on Angie's professional judgment. In this case, the answer is "yes", since FHT clients would be relying on Angie's professional judgment for referral to the grocery store nutrition program. The second step in the framework would be to determine if there is anything that may interfere with Angie's professional judgment to act within the best interests of her clients. (Cohen, 2009)
